# Enhanced anti‐angiogenic activity of novel melatonin‐like agents

**DOI:** 10.1111/jpi.12739

**Published:** 2021-05-13

**Authors:** Su Jung Hwang, Yeonghun Jung, Ye‐Seul Song, Suryeon Park, Yohan Park, Hyo‐Jong Lee

**Affiliations:** ^1^ School of Pharmacy Sungkyunkwan University Suwon Gyeonggi‐do Korea; ^2^ College of Pharmacy and Inje Institute of Pharmaceutical Sciences and Research Inje University Gimhae Gyungnam Korea

**Keywords:** angiogenesis, HIF‐1α, melatonin, melatonin‐like molecules, *N*‐butyryl‐5‐methoxytryptamine, VEGF

## Abstract

Hypoxia‐inducible factor‐1 (HIF‐1) plays an important role in cellular responses to hypoxia, including the transcriptional activation of several genes involved in tumor angiogenesis. Melatonin, also known as N‐acetyl‐5‐methopxytryptamine, is produced naturally by the pineal gland and has anti‐angiogenic effects in cancer through its ability to modulate HIF‐1α activity. However, the use of melatonin as a therapeutic is limited by its low oral bioavailability and short half‐life. Here, we synthesized melatonin‐like molecules with enhanced HIF‐1α targeting activity and less toxicity and investigated their effects on tumor growth and angiogenesis, as well as the underlying molecular mechanisms. Among melatonin derivatives, *N*‐butyryl‐5‐methoxytryptamine (NB‐5‐MT) showed the most potent HIF‐1α targeting activity. This molecule was able to (a) reduce the expression of HIF‐1α at the protein level, (b) reduce the transcription of HIF‐1α target genes, (c) reduce reactive oxygen species (ROS) generation, (d) decrease angiogenesis in vitro and in vivo, and (e) suppress tumor size and metastasis. In addition, NB‐5‐MT showed improved anti‐angiogenic activity compared with melatonin due to its enhanced cellular uptake. NB‐5‐MT is thus a promising lead for the future development of anticancer compounds with HIF‐1α targeting activity. Given that HIF‐1α is overexpressed in the majority of human cancers, the melatonin derivative NB‐5‐MT could represent a novel potent therapeutic agent for cancer.

## 1 INTRODUCTION

In the tumor microenvironment, low oxygen tension (hypoxia) is a major driver of angiogenesis, which can lead to metastasis, chemoresistance, and radioresistance.^1^, ^2^, ^3^ Under hypoxia, hypoxia‐inducible factor‐1α (HIF‐1α) is stabilized and forms a heterodimer with the constitutively expressed HIF‐1β.^4^ This heterodimer, known as HIF‐1, acts as a key regulator of O_2_ homeostasis and allows cancer cells to adapt to hypoxic conditions by the transcriptional activation of downstream genes that regulate glucose metabolism (glucose transporters), angiogenesis [vascular endothelial growth factor (VEGF)], cell proliferation [erythropoietin, insulin‐like growth factor 2(IGF‐2)], and cell survival (galectin‐3, VEGF‐A, Akt).^5^, ^6^ Under normoxic conditions, on the other hand, HIF‐1α is hydroxylated at its proline residue by prolyl hydroxylases (PHDs), which allows it to interact with the von Hippel‐Lindau (VHL) E_3_ ubiquitin ligase complex.^7^ Then, ubiquitylation of HIF‐1α leads to its proteasome‐dependent degradation. In other words, although HIF‐1α is constantly synthesized, it is rapidly degraded under normoxia. HIF‐1α expression is frequently increased in solid cancers, including nonsmall cell lung carcinoma (NSCLC), breast carcinoma, and colorectal carcinoma, and the prognosis of patients with HIF‐1α overexpression is very poor.^2^, ^8^, ^9^ Although there have been many challenges to inhibiting HIF‐1α directly or indirectly, specific targeting of HIF‐1α remains an attractive therapeutic strategy for many solid tumors.

Melatonin (*N*‐acetyl‐5‐methoxytryptamine, **M**) is an endogenous hormone produced by the pineal gland and other organs such as the skin, gastrointestinal tract, and retina in humans and other mammals at night.^10^, ^11^ The concentration of melatonin in the blood peaks between 2 and 5 AM, and its secretion is reduced by light. Accumulating evidence suggests that melatonin is not only an endogenous hormone, but also functions as a regulator of inflammation, redox processes, and hematopoiesis.^12^, ^13^, ^14^, ^15^, ^16^ For example, besides controlling the circadian rhythm, melatonin induces the production of cytokines such as IL‐2, inhibits the production of ROS, primes natural killer cells, and prevents hematopoietic toxicity during chemotherapy.^17^ In addition, melatonin plays an important role in cancer prevention and treatment.^18^ Melatonin has been shown to affect tumor formation and progression through diverse molecular and cellular processes.^19^, ^20^ For example, melatonin significantly inhibits the expression of HIF‐1α and therefore VEGF and thus shows anti‐angiogenic properties.^21^, ^22^

Despite these advantages, melatonin is of limited clinical utility because of its low bioavailability and short half‐life.^23^, ^24^ Although low doses of melatonin can improve sleep disorders such as chronic idiopathic insomnia and Angelman syndrome, high doses (more than 0.3 mg) of melatonin can disrupt the delicate balance of the circadian system and cause serious side effects, including hypothermia.^25^, ^26^ However, the dose of melatonin that results in effective HIF‐1α targeting and anti‐angiogenic effects is higher than that of other anti‐angiogenic drugs. In this study, we synthesized melatonin‐like molecules to overcome these limitations of melatonin while retaining its efficacy. We compared the HIF‐1α targeting and anti‐angiogenic effects of these compounds with those of melatonin and further explored the underlying molecular mechanisms.

## 2 MATERIALS AND METHODS

### 2.1 Chemistry

All reagents from commercial sources were used as sold, without further purification. Organic solvents were concentrated under reduced pressure using a Büchi rotary evaporator. Thin‐layer chromatography (TLC) analyses were performed using Merck precoated TLC plates (silica gel 60 GF254, 0.25 mm). Flash column chromatography was carried out using E. Merck Kieselgel 60 (70‐230 mesh). Melting points were recorded on a Büchi B‐540 melting‐point apparatus. High‐resolution mass spectra (HRMS) were measured on a JEOL JMS‐600W spectrometer. Nuclear magnetic resonance (^1^H‐NMR and ^13^C‐NMR) spectra were measured on a VNMRS‐500 [500 MHz (^1^H), 125 MHz (^13^C)] spectrometer, using CHCl_3_‐*d* as the solvent, and were reported in ppm relative to CHCl_3_ (δ 7.24) for ^1^H‐NMR and relative to the central CDCl_3_ resonance for ^13^C‐NMR. Coupling constants (*J*) in ^1^H‐NMR are in Hz. Fourier‐transform infrared spectra (FT‐IR) were measured on a VARIAN 640‐IR spectrometer. Log *P* (*P*: partition coefficient for *n*‐octanol/water) was determined using ChemBioDraw Ultra software (v14.0) (PerkinElmer, MA, USA). The detailed synthetic procedures and characterizations of melatonin and its derivatives are described in the [Link], [Link].

### 2.2 Cell culture and reagents

Human umbilical vein endothelial cells (HUVECs), NIH‐3T3 embryonic fibroblasts, HeLa cervical cancers, MDA‐MB‐231 breast cancer cells, and HCT116 cancer cells were purchased from the Korean Cell Line Bank (Seoul, Korea), and all cells were maintained at 37°C in a humidified 5% CO_2_ atmosphere. HCT116 cells were cultured in RPMI‐1640 medium supplemented with 10% fetal bovine serum (FBS) and 1% antibiotics (Thermo Fisher Scientific). HUVECs were cultured in 0.3% gelatin‐coated flasks with EGM‐2 medium (Lonza), which contains various endothelial growth factors, including VEGF. To create hypoxic conditions, cells were incubated in a 5% CO_2_ atmosphere with 1% O_2_ balanced with N_2_ in a MIC‐101 hypoxic chamber system (Billups‐Rothenberg Inc). Matrigel, recombinant human VEGF, and antibodies against pVHL and HIF‐1α were purchased from BD Biosciences. CellROX Green was purchased from Thermo Fisher Scientific. Dimethyloxaloylglycine (DMOG) was purchased from Sigma‐Aldrich.

### 2.3 In vitro angiogenesis assays

In vitro angiogenesis assays were performed as described previously.^4^ For tube formation, 100 μL/well of Matrigel (10 mg/mL) was added to 48‐well culture plates and allowed to polymerize for 30 min at 37˚C. HUVECs (5 × 10^4^) were seeded on the Matrigel surface, and tube formation was observed. For migration assays, HUVECs were seeded at a density of 2 × 10^5^/well in 6‐well flat‐bottom plates and a p1000 pipette tip was used to scratch the confluent monolayer in each well. Fresh medium with melatonin or derivatives (0.5 mM) and 1 mM thymidine was added, and the plates were photographed after 24 hours. For Transwell invasion assays, the lower and upper sides of 8‐μm‐porosity polycarbonate filters were coated with 0.5 mg/mL type I collagen and 0.5 mg/mL Matrigel, respectively. Media containing 0.1 mg/mL bovine serum albumin and supplemented with melatonin or derivatives (0.5 mM) was added to the lower compartments, and HUVECs were seeded in the upper compartments. Cell invasion was determined by examining individual filters under an optical microscope at 40x magnification. All experiments were carried out at least 3 times with duplicate independent samples.

### 2.4 In vivo Matrigel plug assays

A mixture of Matrigel and heparin with recombinant bFGF (positive control), recombinant bFGF +melatonin (1.0 mM), or recombinant bFGF +melatonin derivative **2** (1.0 mM) was subcutaneously inoculated into C57BL/6J mice (Hana Bio, Korea). A Matrigel/heparin mixture without bFGF was used as a negative control. The animals were euthanized after 14 days, and the Matrigel plug was removed and photographed. Protocols for animal handling and care were approved by the Institutional Animal Care and Use Committee (IACUC) of Sungkyunkwan University, South Korea.

### 2.5 Cellular uptake of melatonin (**M**) and N‐butyryl‐5‐methoxytryptamine (**2**)

The amount of cellular melatonin (**M**) and *N*‐butyryl‐5‐methoxytryptamine (NB‐5‐MT, **2**) was measured according to the HPLC method of Hevia et al, with some modifications.^27^, ^28^ Briefly, HCT116, HeLa, and MDA‐MB‐231 cancer cells were seeded at a density of 1.0 × 10^5^/mL on 100 mm dishes. After 16 hours, cells were treated with either melatonin or NB‐5‐MT (1.0 mM) for 24 hours and then washed with PBS. After washing, cells were resuspended in 1.0 mL of HPLC‐grade water and kept frozen until processing. A Shimadzu HPLC system with an SPD‐D20A photodiode array (PDA) detector and Nucleosil 100‐5 C18 analytical columns (125 mm length ×4.0 mm i.d., 5 μm particle size; Macherey‐Nagel) was used for quantification of **M** and **2** on the isocratic mode. The mobile phases consisted of water (containing 0.1% formic acid) and acetonitrile (containing 0.1% formic acid) in the proportion of 75:25 (v/v) for **M** and 65:35 (v/v) for **2**. The injection volume was 10 µL with a flow rate of 1.0 mL/min. The column oven temperature was set to 35°C, and the PDA detection wavelength was 220 nm. In HPLC chromatograms, the retention time for the peak was 6.4 min for **M** and 6.5 min for **2**. Five‐point calibration curves for **M** and **2** were established in the range of 0.125‐2.0 mM by serial dilution of the standard stock solutions with MeCN. Samples were lysed by freeze‐thawing. Samples were diluted in MeCN and filtered using a DISMIC 13HP syringe filter (hydrophilicity, 0.2 μm) before HPLC injection. The cellular uptake of **M** and **2** was calculated based on the corresponding calibration curves.

### 2.6 Hypoxia‐induced retinal angiogenesis and tumor xenografts in zebrafish

Wild‐type zebrafish and a transgenic zebrafish line, Tg (*fli1a:EGFP*), were obtained from the Korea Zebrafish Organogenesis Mutant Bank (ZOMB) and maintained in aquaria according to standard procedures on a 10‐hour dark/14‐hour light cycle at 28.5˚C. Mating was induced by the change from dark to light, and embryos were collected at 3‐6 days postfertilization (dpf). Protocols for the handling and care of the zebrafish were approved by the IACUC of Sungkyunkwan University, South Korea.

For hypoxia‐induced retinal angiogenesis, 100 μM dimethyloxaloylglycine (DMOG; Sigma) was used to stimulate abnormal retinal angiogenesis at 2.5 dpf in Tg(*fli1a:EGFP*) zebrafish. Melatonin (**M**) or NB‐5‐MT (**2**) was applied at 4.0 dpf. After 12 hours, lenses were isolated and treated with 3% trypsin in Tris‐HCl (pH 7.8) for 90 min at 37˚C. For each image, 50 optical slices (0.5 µm step z‐stacks) were acquired on a Zeiss LSM 710 confocal microscope system (Carl Zeiss). The stacks were compressed to a maximal projection, and the vessel diameter was determined using ZEN blue software (Carl Zeiss, Germany). Experiments were performed in triplicate and only representative images are shown.

For tumor xenografts, 24 dpf embryos were anesthetized with 0.0016% tricaine and positioned on their right side on a wet 1.0% agarose pad. HCT116 or MDA‐MB‐231 cancer cells were stained with 2 mg/mL DiI diluted in PBS, counted by microscopy, and resuspended in 10% FBS, and 200 cells were injected into the center of the yolk sac of each embryo using an injector (PV820 pneumatic picopump; World Precision Instruments, FL, USA) equipped with borosilicate glass capillaries. Injected embryos were transferred to 96‐well plates containing the drugs of interest diluted in 200 mL E3 media and maintained at the preselected incubation temperature. The numbers of embryos exhibiting cancer cell dissemination from the injection site were counted by upright microscopy (Carl Zeiss Microscope, Germany). To quantify the proliferation of cancer cells in zebraﬁsh larvae, zebrafish embryos were used at 1 and 4 days postinjection (dpi). The cells were imaged by inverted ﬂuorescent microscopy at 20x magnification using ImagePro Plus software (Media Cybernetics, Silver Spring, MD). For quantitative measurement of tumor xenografts, 0.5 µm step z‐stacks (50‐200 µm in depth) were acquired using a Z1 fluorescence microscope (Carl Zeiss, Germany). Then, Axiovision Rel 4.8 software (Carl Zeiss) was used to measure the width and length of each xenograft. The tumor volume in mm^3^ was calculated using the formula: volume = (width)^2^ × length/2.

### 2.7 Statistical analysis

All measurements were performed in a blinded fashion. Data were expressed as the mean ±SEM. A *P* value of <.05 was considered statistically significant. Student's *t* tests were used when 2 groups were compared. One‐way ANOVA followed by a Tukey's multiple comparisons test was used when 3 or more groups were compared. Statistical analyses were performed with GraphPad Prism (v6.0) (GraphPad Software).

## 3 RESULTS

**FIGURE 1:**
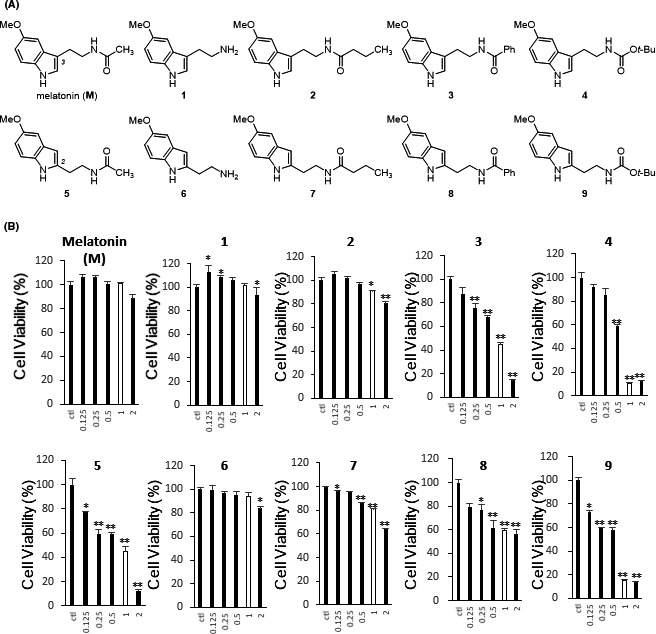
Structure and IC_50_ of melatonin‐like molecules. A, The molecular structures of melatonin (**M**) and its derivatives **1**‒**9**. B, The effect of **M** and **1**‒**9** on cell viability in HCT116 human colon cancer cells. White bars indicate the cell viability when treated with compound at 1.0 mM. Values are expressed relative to vehicle‐treated cells, normalized to 100% ± the standard deviation (SD), and were obtained from 3 independent experiments. *P <.05, **P <.01, and ***P <.001 in vehicle vs. compound treatment. The IC_50_ values of **M** and **1**‒**9** in HCT116 human colon cancer cells were 4.48, 6.25, 5.60, 0.81, 0.56, 0.56, 8.51, 3.67, 2.87, and 0.41 mM, respectively

Melatonin‐like molecules were synthesized through 2 different methods. In Supplementary Figure S1A, reactions between the commercially available compound **1** and an appropriate RCOX generated melatonin (**M**) and melatonin homologues **2** − **4,** which have an aminoethyl branch at C3 of the indole, in 1 step (73 − 93% yield). To synthesize melatonin isomers **5** − **9**, in which the branch has migrated from C3 to C2, we followed Spadoni's protocol,^29^ as shown in Supplementary Figure S1B. The indole carboxylic acid **10** was changed to acid chloride with SOCl_2_, and then the addition of dimethyl amine synthesized tertiary amide **11** (81% yield). Amide **11** was reduced to a tertiary amine by LiAlH_4_, and additional methylation of the tertiary amine gave quaternary ammonium **12** (73% yield). Cyanide anion substitution of the quaternary ammonium **12**, followed by reduction of the cyano group, afforded primary amine **6** (72% yield). Finally, melatonin isomers **5** and **7** − **9** were synthesized by adding proper RCOX to primary amine **6** (72 − 99% yield). Figure 1A shows the structure of melatonin (**M**) and its derivatives **1** − **9**.

Previously, Kim et al reported that melatonin inhibits tumor progression by suppressing angiogenesis mediated by HIF‐1α.^22^ Thus, we compared the toxicity and efficacy of melatonin‐like molecules to find novel therapeutic agents that inhibit angiogenesis without cellular toxicity. Melatonin‐like molecules have been reported to have cytoprotective and radioprotective effects.^29^, ^30^ First, we compared the cytotoxic effects of melatonin derivatives on HCT116 human colon cancer cells. After treatment with melatonin (**M**) or melatonin‐like compounds **1**‒**9** over a 48‐hr period, cell viability was determined by MTS assay. As shown in Figure 1B, M did not show cytotoxicity at concentrations up to 1.0 mM and decreased cell viability to 93.62% of the control at 2.0 mM (IC_50_ = 4.48 mM). The IC_50_ values of melatonin derivatives **1**‒**9** varied from 0.81 to 8.51 mM. Previously, 1.0 mM melatonin was shown to inhibit HIF‐1α‐mediated angiogenesis without cellular toxicity.^22^ Like melatonin, melatonin‐like molecules **1**, **2**, **6**, and **7** did not affect cell viability at a concentration of 1.0 mM, but compounds **3**, **4**, **5**, **8**, and **9** exhibited significant cytotoxicity at 1.0 mM. Based on the IC_50_ values, some derivatives with relatively high toxicity (**3**, **4**, **5**, **8**, and **9**) were excluded from further analysis, and the remaining derivatives (**1**, **2**, **6**, and **7**) were analyzed further.

**FIGURE 2:**
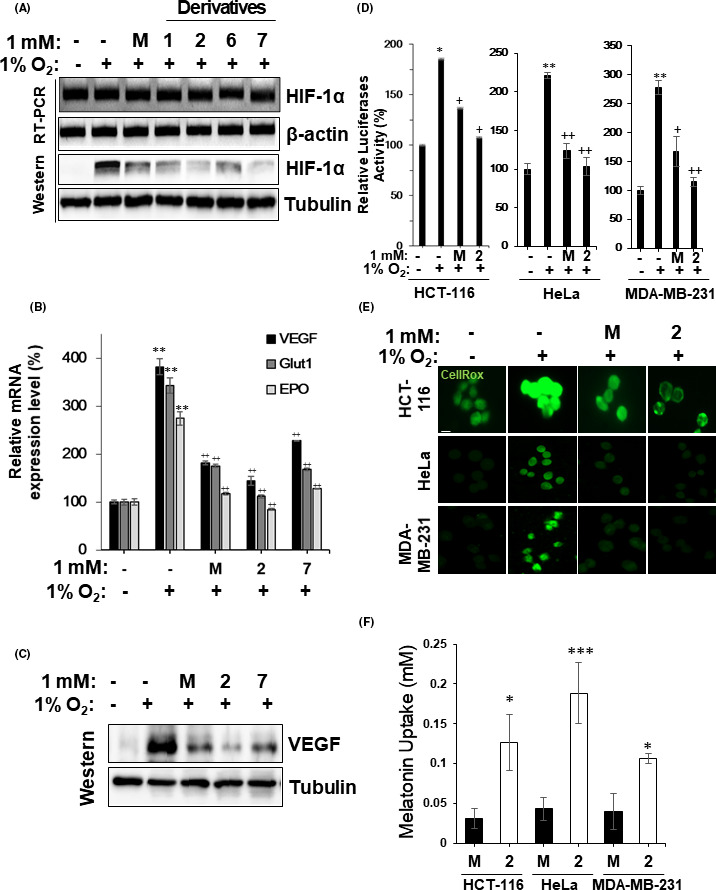
NB‐5‐MT (2) suppresses HIF‐1‐mediated transactivation and ROS generation through enhanced cellular uptake. (A‐B) Under hypoxia, HCT116 cells were treated with 1 mM of melatonin (**M**), or its derivatives **1**, **2**, **6**, and **7** for 4 hr The expression of HIF‐1α and VEGF was analyzed by RT‐PCR and western blot. A, The effect of **M**, **1**, **2**, **6**, and **7** on HIF‐1α mRNA and protein expression under hypoxic conditions. B, The effect of **M**, **2**, and **7** on VEGF, Glut1, and EPO mRNA expression under hypoxic conditions. β‐actin was used as a loading control. C, The effect of **M**, **2**, and **7** on VEGF protein expression under hypoxic conditions. Tubulin was used as the loading control. Experiments were performed in triplicate, and representative images are shown. D, The effect of derivative **2** on HIF‐1α‐mediated transactivation. HCT116, HeLa, and MDA‐MB‐231 cells were transfected with pSV40promoter‐EpoHRE‐Luc and then treated with either 1 mM of **M**, or **2**. Values are expressed relative to vehicle‐treated cells, normalized to 100. Values shown are the means ± SD and were obtained from 3 independent experiments. *P <.05 in vehicle vs. hypoxia; +*P* <.05, hypoxia vs. hypoxia plus **M** or **2** treatment. E, The effect of **2** on oxidative stress in HCT116, HeLa, and MDA‐MB‐231 cells. Cells were treated with either 1 mM of **M**, or **2** under hypoxia. Intracellular ROS were stained with a fluorescent dye (CellROX Green Reagent). Cell nuclei were counterstained with Hoechst 33 362 (blue). Experiments were performed in triplicate and representative images are shown. F, Cellular uptake of **2** in HCT116, HeLa, and MDA‐MB‐231 cells. Values are the means ± SD and were obtained from 3 independent experiments. *P <.05

Next, we investigated the effect of melatonin derivatives **1**, **2**, **6**, and **7** on the expression of HIF‐1α, which is essential for tumor progression. When HCT116 cells were treated with melatonin, HIF‐1α expression under hypoxic conditions dramatically decreased at the protein level but not at the mRNA level (Figure 2A). Similarly, compounds **1**, **2**, **6**, and **7** did not affect HIF‐1α mRNA levels but reduced the HIF‐1α protein level significantly. In particular, compounds **2** and **7** showed a much stronger inhibitory effect on HIF‐1α protein levels than melatonin. The suppressive effects of **2** and **7** on HIF‐1α‐mediated gene transcription were investigated based on changes in the mRNA levels of the HIF‐1α downstream targets VEGF, glucose transporter‐1 (Glut1), and erythropoietin (EPO), which are crucial for tumor progression and angiogenesis.^31^, ^32^ When compared with melatonin, NB‐5‐MT (**2**) strongly reduced the levels of VEGF, Glut1, and EPO mRNA, but **7** did not (Figure 2B). The inhibitory effect of NB‐5‐MT (**2**) on VEGF expression was confirmed at the protein level (Figure 2C). We next compared the potential of melatonin homologue **2**, *N*‐butyryl‐5‐methoxytryptamine (NB‐5‐MT), as a novel melatonin replacement candidate. Because melatonin (**M**) is known to affect HIF‐1α expression as well as HIF‐1α transcriptional activity, we examined the inhibitory effect of NB‐5‐MT (**2**) on the transcriptional activity of HIF‐1α by luciferase reporter assay in HCT116, HeLa, and MDA‐MB‐231 cells. As shown in Figure 2D, NB‐5‐MT (**2**) inhibited the transcriptional activity of HIF‐1α much more strongly than **M** under hypoxia. In addition, the inhibitory effect of NB‐5‐MT (**2**) on the expression levels of HIF‐1α and its target genes was confirmed in HeLa and MDA‐MB‐231 cells (Supplementary Figure S2). Since melatonin has strong antioxidant capacity, we compared the suppressive effect of NB‐5‐MT (**2**) with that of **M** using a cell‐permeable fluoroprobe for measuring oxidative stress (CellROX Green Reagent) and found that NB‐5‐MT (**2**) showed much stronger antioxidative effects than melatonin in HCT116, HeLa, and MDA‐MB‐231 cells (Figure 2E). To investigate the mechanism through which NB‐5‐MT (**2**) inhibited HIF‐1α transcription and oxidative activity more strongly than melatonin, we analyzed the partition coefficients (log *P*‐values) of melatonin and its derivatives. Most of the melatonin‐like molecules (except **5** and **6**) showed much higher log *P*‐values than melatonin (log *P*_M_ = 0.71), suggesting that these derivatives are expected to be cytotoxic due to excessive intracellular flux (Supplementary Figure S3). On the other hand, melatonin derivatives **2** and **7,** which had log *P*‐values that were 2.3‐2.5 times higher than that of melatonin, exhibited equal or greater effects on HIF‐1α‐mediated transcription than melatonin without severe cytotoxicity. In fact, even though the cellular influx of NB‐5‐MT (**2**) was more than 4 times as fast as that of the melatonin in HCT‐116 cells (Figure 2F), there was no significant increase in toxicity. These results were also confirmed in HeLa and MDA‐MB‐231 cells. Taken together, these results indicate that NB‐5‐MT (**2**) has greater effects on HIF‐1α‐mediated transcription and ROS generation than melatonin due to its enhanced cellular uptake.

**FIGURE 3:**
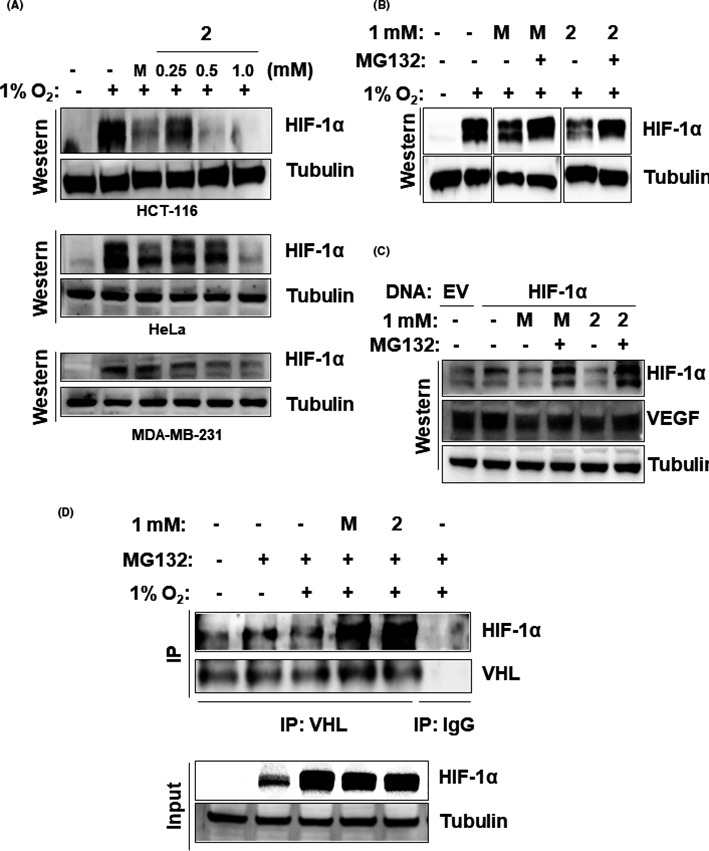
NB‐5‐MT (2) reduces HIF‐1α stability through pVHL binding. A, The dose dependence of **2** on HIF‐1α expression. Under hypoxia, HCT116, HeLa, and MDA‐MB‐231 cells were treated with various concentrations (0.125‐1.0 mM) of **2** for 4 hr HIF‐1α expression was examined by western blot analysis. B, The effect of **2** on the stability of endogenous HIF‐1α. Under hypoxia, HCT116 cells were incubated with or without 1.0 mM of **2** in the presence of the proteasome inhibitor MG132 for 4 hr (C) The effect of **2** on the stability of exogenous HIF‐1α and the expression of VEGF. Empty vector (EV) or HIF‐1α expression plasmid was transiently transfected in HCT116 cells. After 24 h, HCT116 cells were incubated with or without 1.0 mM of **2** in the presence of the proteasome inhibitor MG132 for 4 hr (D) The effect of **2** on the binding between HIF‐1α and pVHL. Under hypoxia, HCT116 cells were incubated with or without 1.0 mM of **2** in the presence of the proteasome inhibitor MG132 for 4 hr, and then 500 μg of total protein was immunoprecipitated with either anti‐IgG or anti‐VHL antibodies. Western blotting was performed with anti‐HIF‐1α and anti‐pVHL antibodies

Next, we examined how NB‐5‐MT (**2**) affects HIF‐1α protein levels by examining changes in HIF‐1α protein stability. First, we confirmed that the downregulation of HIF‐1α protein in response to NB‐5‐MT (**2**) was dose‐dependent in HCT116, HeLa, and MDA‐MB‐231 cells (Figure 3A), suggesting that it affects HIF‐1α protein stability. To confirm this, HCT116 cells were co‐treated with NB‐5‐MT (**2**) and MG132, a proteasome inhibitor, under hypoxia, and the HIF‐1α protein levels were measured. Co‐treatment with NB‐5‐MT (**2**) and 5 μM MG132 restored the HIF‐1α level to that of hypoxia‐exposed cells without NB‐5‐MT (**2**) treatment (Figure 3B). To verify this, the effectiveness of NB‐5‐MT (**2**) under overexpression conditions was investigated using a HIF‐1α expression vector. As shown in Figure 3C, HCT‐116 cells transfected with HIF1‐α expression vectors showed the increased level of HIF1‐α and VEGF compared to that of empty vector (EV)‐transfectants under normoxic condition. However, NB‐5‐MT(2) notably decreased the increase in HIF1‐α and VEGF proteins which are induced by HIF1‐α transfection. In addition, MG132, a proteasome inhibitor, blocked the proteasomal degradation of HIF‐1 protein mediated by NB‐5‐MT(2), suggesting that NB‐5‐MT(2) affects HIF‐1α protein stability. Both melatonin (**M**) and NB‐5‐MT (**2**) showed similar proteasome‐dependent degradation of HIF‐1α protein. At a normal oxygen concentration, HIF‐1α is hydroxylated by prolyl‐4‐hydroxlyases (PHDs) and then interacts with Von‐Hippel‐Lindau (pVHL), an E3 ubiquitin ligase, resulting in poly‐ubiquitination and proteasomal degradation. In previous studies, it was reported that melatonin restored PHD activity,^22^ so we investigated the effect of NB‐5‐MT on the activity of PHD using a HIF‐1α‐pVHL binding assay. When HCT116 cells were exposed to hypoxia, binding of HIF‐1α and pVHL was decreased (Figure 3D). However, binding of HIF‐1α and pVHL under hypoxia was dramatically increased by NB‐5‐MT (**2**), actually reaching the level of HIF‐1α‐pVHL binding in normoxia (Figure 3D). Interestingly, the increase in PHD activity by NB‐5‐MT (**2**) appeared to be much stronger than that of melatonin. These results suggest that NB‐5‐MT (**2**) reduces hypoxia‐induced HIF‐1α stability through pVHL binding.

**FIGURE 4:**
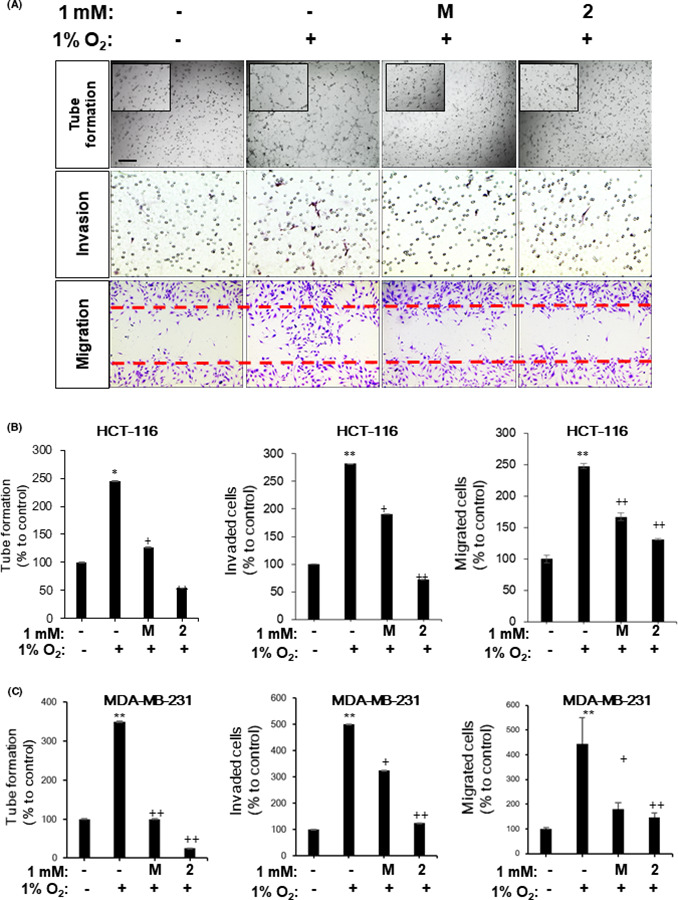
NB‐5‐MT (2) suppresses hypoxia‐mediated angiogenesis in vitro. (A‐B) Under hypoxia, HCT116 cells were incubated with 1 mM of melatonin (**M**) or **2** for 16 hr Conditioned medium (CM) was collected from HCT116 cells or MDA‐MB‐231 cells and added to HUVECs for angiogenesis assays. (A) Inhibitory effect of **2** on angiogenesis in human umbilical vein endothelial cells (HUVECs) in vitro. Representative images of tube formation (top), invasion (middle), and migration (bottom) assays from 3 independent experiments. (B) Relative anti‐angiogenic effect of **2** using the conditioned media of HCT116 cells. Graphs of in vitro tube formation (left), invasion (middle), and migration (right). (C) Relative anti‐angiogenic effect of **2** using the conditioned media of MDA‐MB‐231 cells. Graphs of in vitro tube formation (left), invasion (middle), and migration (right). Data are the mean ± SD from 3 independent experiments. *P <.05 and **P <.01, vehicle vs. hypoxia; +*P* <.05 and ++*P* <.01, hypoxia vs. hypoxia plus compound treatment

To investigate the anti‐angiogenic activity of NB‐5‐MT (**2**) in vitro, HCT116 cells were incubated with either melatonin (**M**) or NB‐5‐MT (**2**) under hypoxia for 16 hr Then, conditioned medium (CM) from the HCT116 cells was applied to HUVECs for in vitro angiogenesis assays. As shown in Figure 4A, CM from hypoxia‐treated tumor cells increased tube formation, invasion, and migration, but CM from hypoxia‐treated tumor cells treated with NB‐5‐MT (**2**) inhibited these effects. The inhibitory effect of NB‐5‐MT (**2**) on angiogenesis seemed to be much stronger than that of melatonin (Figure 4B). These anti‐angiogenic effects of **2** were reproduced equally in MDA‐MB‐231 cells (Figure 4C). Taken together, these data indicate that NB‐5‐MT (**2**) suppresses hypoxia‐mediated angiogenesis in vitro.

**FIGURE 5:**
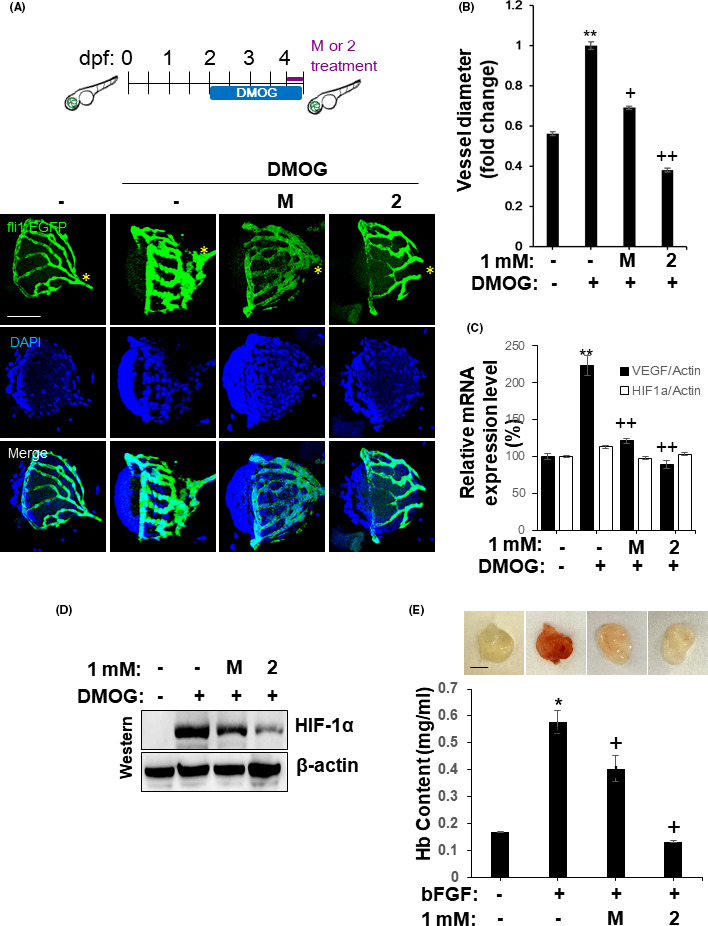
NB‐5‐MT (2) suppresses angiogenesis in vivo. (A‐B) Inhibitory effect of **2** on retinal vascularization in DMOG‐treated Tg(fli1a:EGFP) zebrafish larvae. A, Representative images of retinal vascularization. DMOG‐mediated retinal vascularization was decreased by treatment with 1 mM of **2**. Nuclei were stained with 1 μg/ml DAPI (PBS). Scale bar, 100 μm. B, Quantification of vessel diameters in zebrafish retinas. Data are expressed as the mean ± SD (n = 20). **P <.01, vehicle vs. DMGF; +*P* <.05 and ++*P* <.01, DMOG vs. DMOG plus compound. C, Inhibitory effect of **2** on VEGF and HIF‐1α mRNA expression in DMOG‐treated zebrafish larvae. D, Inhibitory effect of **2** on HIF‐1α protein expression in zebrafish larvae. (E) Inhibitory effect of **2** in an in vivo Matrigel plug assay. Matrigel plugs were prepared with bFGF alone, bFGF plus melatonin, or bFGF plus **2**, and implanted into C57/BL6 mice. The mice were euthanized 15 days after implantation, and the plugs were recovered. Representative images of macroscopic Matrigel plugs (top panels) from 3 independent experiments. Scale bar, 0.5 mm. Hemoglobin levels in the Matrigel plugs (bottom) were determined using Drabkin's solution. The hemoglobin content was normalized to the weight of the plug. Data are expressed as the mean ± SD (n = 6). *P <.05, vehicle vs. bFGF; +*P* <.05, bFGF vs. bFGF plus compound

To further extend this observation, we investigated the anti‐angiogenic activity of NB‐5‐MT (**2**) in 2 different in vivo models, a dimethyloxalylglycine (DMOG)‐induced hypoxia model in zebrafish larvae and a mouse Matrigel plug assay.^4^, ^33^ First, transgenic zebrafish larvae at 2.5 days postfertilization (dpf) were challenged with 100 μmol/L DMOG, which stabilizes HIF‐1α, thus mimicking hypoxia‐mediated angiogenesis. DMOG increased the vessel diameter in zebrafish embryos dramatically. However, melatonin (**M**) and NB‐5‐MT (**2**) abolished the DMOG‐induced increase in vessel diameter (Figure 5A and B). Embryos treated with NB‐5‐MT (**2**) were much thinner than the vessels of melatonin‐treated embryos. We next confirmed whether the inhibitory effect of NB‐5‐MT (**2**) derived from inhibition of the HIF‐1/VEGF axis in zebrafish. NB‐5‐MT (**2**) significantly decreased HIF‐1α protein levels and VEGF mRNA levels, but not HIF‐1α mRNA levels, in DMOG‐treated zebrafish (Figure 5C and D), as well as in zebrafish under hypoxia (Supplementary Figure S4A and S4B). Next, we performed a Matrigel plug assay in mice. Matrigel plugs mixed with bFGF (200 ng/mL) and implanted into mice appeared dark red when they were recovered after 15 days (Figure 5E, top). However, plugs with Matrigel alone or Matrigel mixed with NB‐5‐MT (**2**) were transparent, which indicates less blood vessel formation. To quantify vessel formation inside the Matrigel plugs, the hemoglobin (Hb) content was analyzed. Melatonin (**M**) reduced the Hb content by 29.9%, while NB‐5‐MT (**2**) decreased the Hb content by 77.5% (Figure 5E, bottom). This suggests that the inhibitory effect of NB‐5‐MT (**2**) on angiogenesis was much greater than that of melatonin. Collectively, these data suggest that NB‐5‐MT (**2**) suppresses angiogenesis in vivo by blocking the HIF‐1/VEGF axis.

**FIGURE 6:**
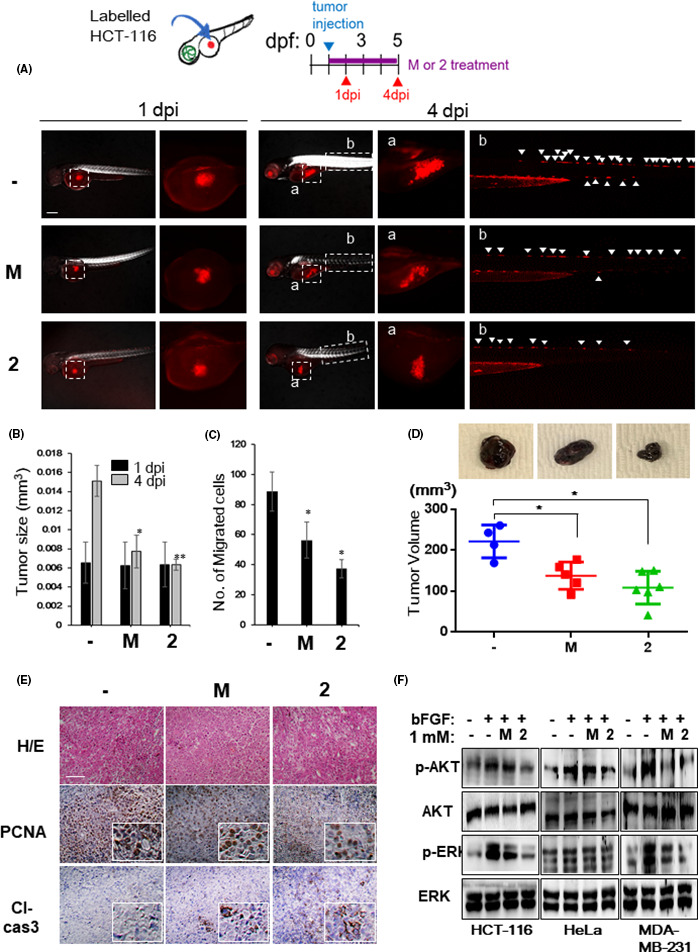
NB‐5‐MT (2) inhibits tumor growth and invasion in a zebrafish xenograft model in vivo. Human HCT116 cancer cells were injected into the abdominal perivitelline space of zebrafish larvae at 2 days postfertilization (dpf) and treated with vehicle, melatonin (**M**), or NB‐5‐MT (**2**). Photographs were taken at 1 and 4 dpi, and the xenograft size and the number of cancer cells that invaded into nearby tissues were quantified in 20 larvae/group. (A) Representative images from 20 fish/group at 1 and 4 dpi. Scale bar, 200 μm. Regions of interest (labeled a, b) are indicated on overview images of the xenografts in the yolk sac and migrated cancer cells in the trunk, respectively. White arrowheads indicate the migrated cancer cells. (B) The size of the tumor xenografts was analyzed quantitatively at 1 and 4 dpi. C, The average number of migrating cancer cells at 4 dpi. The data represent the mean ± SD (n = 3). *P <.05 and **P <.01, vehicle vs. compound treatment. (D) The effects of **2** on the tumor growth using mouse xenograft model of murine melanoma B16F10 cells. Top, the representative images of the tumor growth. (E) Proliferating cell nuclear antigen (PCNA) and cleaved caspase‐3 (Cl‐cas3) expressions were analyzed by immunohistochemical analysis in mouse xenograft tumors. Scale bar, 500 μm. (F) The effect of **2** on AKT and ERK signaling in HCT116, HeLa, and MDA‐MB‐231 cells. Representative western blot showing phosphorylated AKT (p‐AKT), total AKT, phosphorylated ERK (p‐ERK), and total ERK levels

Finally, we wanted to verify whether NB‐5‐MT (**2**) can inhibit tumor growth and invasion in vivo using a zebrafish xenograft model. Since HCT116 cancer cells are known to migrate in zebrafish larvae, we investigated the effect of NB‐5‐MT (**2**) in this model. HCT116 cells were injected into the yolk sacs of zebrafish larvae, and the larvae were treated with vehicle, melatonin (**M**), or NB‐5‐MT (**2**). At 1.0 days postinjection (dpi), there was no significant difference in tumor size between vehicle‐treated, **M**‐treated, and NB‐5‐MT (**2**)‐treated larvae (Figure 6A and B). At 4.0 dpi, however, melatonin and NB‐5‐MT (**2**) decreased the tumor size by 94.8% and 95.8%, respectively (Figure 6A and B). In addition, melatonin and NB‐5‐MT (**2**) decreased the tumor migration by 36.5% and 57.9%, respectively (Figure 6A and C). These effects of NB‐5‐MT (**2**) on tumor growth were confirmed consistently in MDA‐MB‐231 xenograft tumors (Supplementary Fig. S5). To verify that the inhibitory effect of NB‐5‐MT (**2**) was due to its toxicity, additional toxicity tests were performed in 2 normal cell lines (Wi38 lung fibroblast cells and NIH‐3T3 embryonic fibroblast cell), 2 cancer cell lines (HeLa and MDA‐MB‐231 cells), and zebrafish. Melatonin and NB‐5‐MT (**2**) did not exhibit cytotoxicity up to 1 mM, regardless of cell type (Supplementary Figure S6A and S6B). Up to 1 mM of NB‐5‐MT(**2**), zebrafish larvae had a normal heartbeat rate (Supplementary Figure S6C) and normal phenotype without death and hemorrhage (Supplementary Figure S6D). These data suggest that NB‐5‐MT(2) did not have any toxicities both in vivo and in vitro. Furthermore, we investigated the inhibitory effect of NB‐5‐MT (**2**) on tumor growth using the mouse xenograft model. As shown in zebrafish xenografts, NB‐5‐MT (**2**) showed notable suppressive effect on tumor growth in B16F10 tumor xenograft (Figure 6D). Next, we examined whether NB‐5‐MT (**2**) affects the level of proliferating cell nuclear antigen (PCNA) and cleaved caspase‐3 in xenograft tissues. NB‐5‐MT (**2**)‐treated mice showed much weaker PCNDA staining and stronger cleaved caspase‐3 in tumor tissues, compared to those of control (Figure 6E), suggesting that NB‐5‐MT (**2**) inhibits tumorigenesis through increased apoptosis and decreased proliferation in mouse xenograft model. To further assess how NB‐5‐MT (**2**) inhibited tumor growth and invasion, we investigated whether NB‐5‐MT (**2**) affected the activation of AKT and/or extracellular‐related kinase (ERK), 2 proteins known to play an important role in tumor growth and invasion. As shown in Figure 6F, NB‐5‐MT (**2**) significantly reduced the levels of both phosphorylated AKT and ERK in HCT116, HeLa, and MDA‐MB‐231 cells. Taken together, these results indicate that NB‐5‐MT (**2**) inhibits tumor growth and invasion in vivo by blocking the HIF‐1/VEGF axis.

## 4 DISCUSSION

Melatonin (**M**) is an endogenous hormone that has beneficial antioxidant and anti‐inflammatory properties and promotes sleep, when it is used at low concentrations.^34^, ^35^, ^36^, ^37^, ^38^, ^39^ However, melatonin can cause serious side effects, particularly when used in combination with other drugs. For example, there is concern that the use of artificial melatonin for the treatment of jet lag may damage the retina, as it has been reported that systemic administration of melatonin increases light‐induced retinal damage in rats.^40^ In addition, administration of melatonin to children under the age of 13 is prohibited because it can be harmful to normal growth and hormonal balance. Melatonin use may promote inflammation in individuals with autoimmune disease and may increase bleeding in conjunction with blood thinners. Another disadvantage of melatonin is its low and variable bioavailability.^41^, ^42^, ^43^, ^44^ For instance, DeMuro et al reported that melatonin shows very low bioavailability in commonly used dosages (from 2.0 to 4.0 mg) because of poor oral absorption, extensive first‐pass effect, or a combination of factors.^41^ These limitations prompted us to develop new melatonin‐like molecules with enhanced uptake, improved efficacy, and lower toxicity.

Hypoxia causes heterogeneity within the solid tumor microenvironment and is indispensable for tumor cell survival and metastatic potential. Thus, hypoxia is an independent marker of poor prognosis in many types of cancers, including breast, nonsmall cell lung, head and neck, ovarian, and cervical cancer. Usually, hypoxia results from an imbalance between demand and availability of oxygen and nutrients by rapidly proliferating tumor cells and an inadequate, dysfunctional blood supply. Tumor cells sense and respond to hypoxic conditions through activation of the transcription factor HIF‐1, which consists of HIF‐1α and HIF‐1β subunits. HIF‐1 induces the expression of various downstream genes, including VEGF. Because VEGF directly promotes the abnormal growth of blood vessels, VEGF is an essential target in vascular disease as well as cancer. The HIF‐1/VEGF axis is one of the most important regulators of hypoxia‐induced angiogenesis, and its aberrant regulation is associated with hypoxic solid tumors. HIF‐1α and its downstream target VEGF are therefore major targets of many anticancer therapies. It has also been reported that HIF inhibition increases the effectiveness of conventional angiogenesis inhibitors. For example, HIF inhibitors have been reported to significantly increase the anticancer effect of angiogenic inhibitors such as sunitinib or bevacizumab on combined administration.^45^ Therefore, further study is needed to investigate whether NB‐5‐MT (**2**) is effective alone or combined with other anti‐angiogenic inhibitors and examine the underlying molecular mechanisms.

In this study, we synthesized melatonin and derivatives of melatonin with different log *P*‐values and compared their cytotoxicity and ability to inhibit HIF‐1 transcriptional activity with that of melatonin. Melatonin (**M**) and its derivatives **1**, **2**, **6**, and **7** had log *P*‐values of between 0.65 and 1.78, and had low or no toxicity, but derivatives with log *P‐*values that were outside this range (**3**, **4**, **5**, **8**, and **9**) showed very high toxicity. Generally, as the log *P*‐value increases, the uptake of the drug through the cell membrane increases, and thus it is assumed that derivatives with high log *P*‐values will show severe toxicity due to rapid cellular entry. However, even though NB‐5‐MT (**2**) has a log *P*‐value 2.5 times higher than that of melatonin, its entry into the cell is increased compared with melatonin but not its toxicity.

As described above, melatonin has low bioavailability when administered orally. One of the various attempts to overcome this is drug delivery through other routes. For example, recent studies have shown that transdermal administration of melatonin has good efficacy for local applications because melatonin is absorbed slowly and remains in the skin.^42^ Since we have synthesized melatonin‐like molecules **1**‒**9** with various log *P*‐values, we plan to further investigate their absorption through the skin and their efficacy against skin cancer.

Many preclinical and clinical studies have shown the beneficial effects of melatonin against cancer as a single therapeutic agent or in combination with other therapies. Because melatonin interacts with many different target proteins, it could regulate 8 or more of the hallmarks of cancer, including sustained proliferation, evasion of growth suppression, and angiogenesis.^46^ Here, we found that the melatonin derivative NB‐5‐MT has similar mechanisms of action as melatonin. For example, it decreased the synthesis of HIF‐1α through AKT/ERK downregulation and promoted the destabilization of HIF‐1α protein by restoring PHD activity under hypoxia. The inhibitory effect of NB‐5‐MT(**2**) on HIF‐1α might be not restricted to HIF‐mediated angiogenesis: NB‐5‐MT(**2**) could also potentially affect other HIF‐mediated processes, such as cellular circadian rhythms.^47^ Many studies have reported that hypoxia, as a signal for the molecular clock, disturbs the normal circadian rhythm in cancer, and there is a bidirectional relationship between the HIF‐1α signaling pathway and the core clock genes that control the circadian rhythm.^48^ However, when treated with the melatonin receptor 1 and 2 (MT1/2) antagonist (luzindole), the inhibitory effects on HIF‐1, anti‐tumorigenic, and anti‐angiogenic effects by NB‐5‐MT(**2**) were not affected (Supplementary Figure S7). Therefore, it is expected that NB‐5‐MT(**2**) could work through different pathways that are probably independent of MT1/2. For example, HIF‐1α acts as a circadian clock disruptor by interfering with core clock genes such as brain and muscle ARNT‐like 1 (*BMAL1*); BMAL1 also binds to the HIF‐1α promoter, thus controlling the expression of HIF‐1α. Given that HIF‐1α regulates cells’ circadian rhythms, and circadian rhythms affect HIF‐1α activity, it would be meaningful to investigate the effects of melatonin or its derivative, NB‐5‐MT, on HIF‐1α‐mediated regulation of circadian genes.

Our current data identify the melatonin‐like compound NB‐5‐MT (**2**) as a novel therapeutic agent with improved HIF‐1/VEGF targeting and anti‐angiogenic effects compared with melatonin. Further studies are required to delineate the exact mechanism of action of NB‐5‐MT (**2**), for example, whether and how blood vessels in hypoxic regions regress in NB‐5‐MT (**2**)‐treated tumor masses, and whether **2** has improved bioavailability, through pharmacokinetic studies in vivo. Our present studies provide a proof of principle for the development of NB‐5‐MT as an anti‐angiogenic agent.

## CONFLICT OF INTEREST

All authors of this manuscript declare that they have no conflicts of interest.

## AUTHOR CONTRIBUTIONS

S.J.H. conducted in vitro assays and performed or contributed to in vivo experiments. Y.J. designed and synthesized melatonin derivatives. Y.S.S. contributed to in vivo experiments, and S.P. prepared the synthetic strategy. Y.P. and H.J.L. conceived and designed the study and wrote the manuscript.

## Supporting information

Supplementary Material

Supplementary Material

## Data Availability

The data that support the findings of this study are available from the corresponding author upon reasonable request.
