# Supplementary material for: Enhanced anti‐angiogenic activity of novel melatonin‐like agents
Source: J Pineal Res. 2021 May 13;71(1):e12739. doi: 10.1111/jpi.12739 (PMC8365647; doi:10.1111/jpi.12739)
Supplement: Supplementary file 1 — Supplementary Material [file JPI-71-e12739-s001.docx]

***Supporting Information***

**Enhanced anti-angiogenic activity of novel melatonin-like agents**

**(Running Title: The novel anti-angiogenic melatonin-like agents)**

Su Jung Hwang^1,3^ , Yeonghun Jung^2,3^, Ye Seul Song^1^, Suryeon Park^2^, Yohan Park^2,^***, Hyo-Jong Lee^1,^***

*^1^School of Pharmacy, Sungkyunkwan University, 2066 Seobu-ro, Jangan-gu, Suwon, 16419, Republic of Korea; ^2^College of Pharmacy and Inje Institute of Pharmaceutical Sciences and Research, Inje University, 197 Inje-ro, Gimhae, Gyungnam 50834 South Korea. ^3^These authors contributed equally to this work.*

e-mail: yohanpark@inje.ac.kr (Y. Park), hjlee@inje.ac.kr (H-J. Lee).

**Supplementary Figures**

***
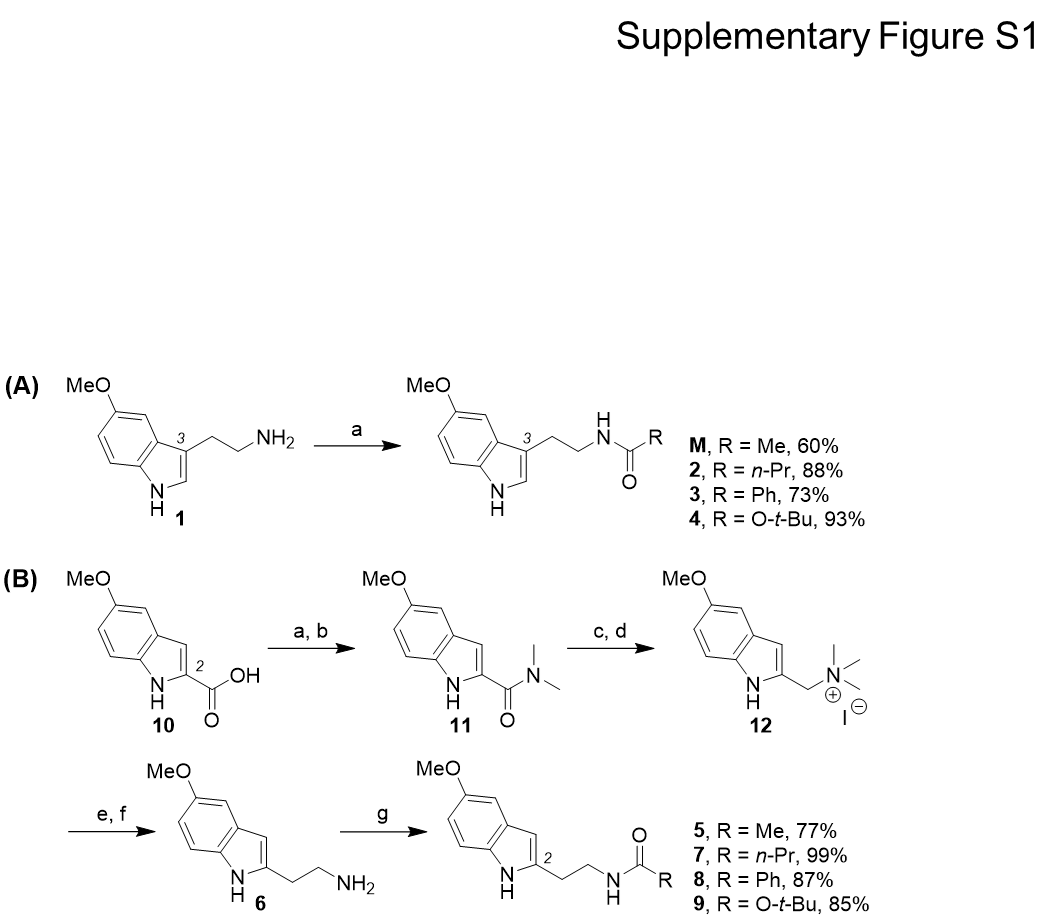
***

***
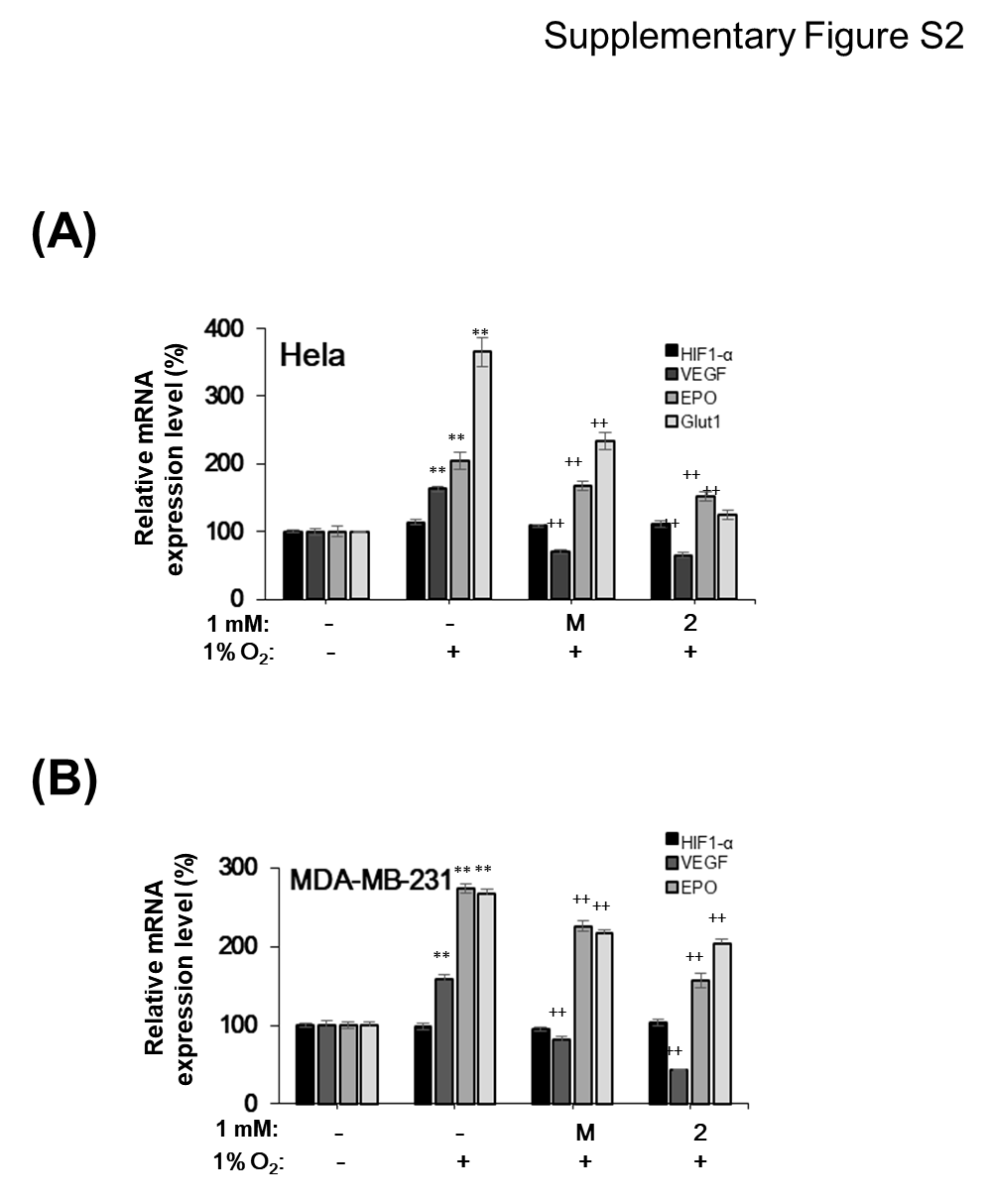
***

***
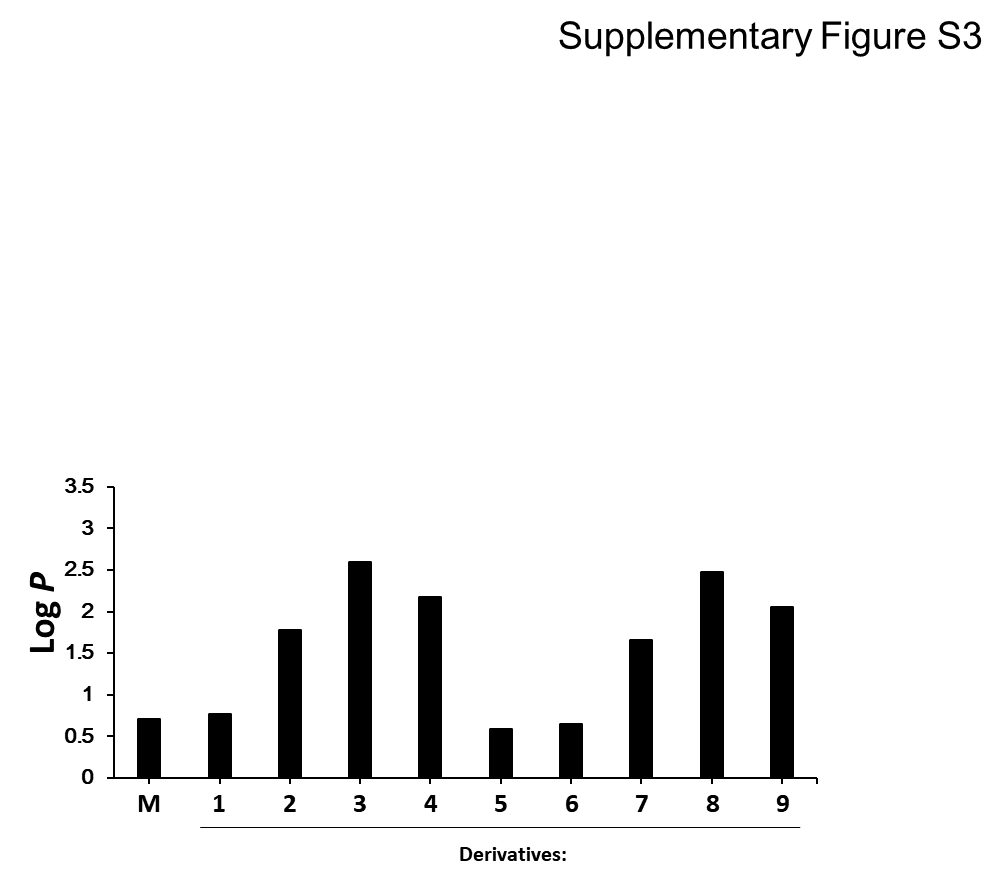
***

***
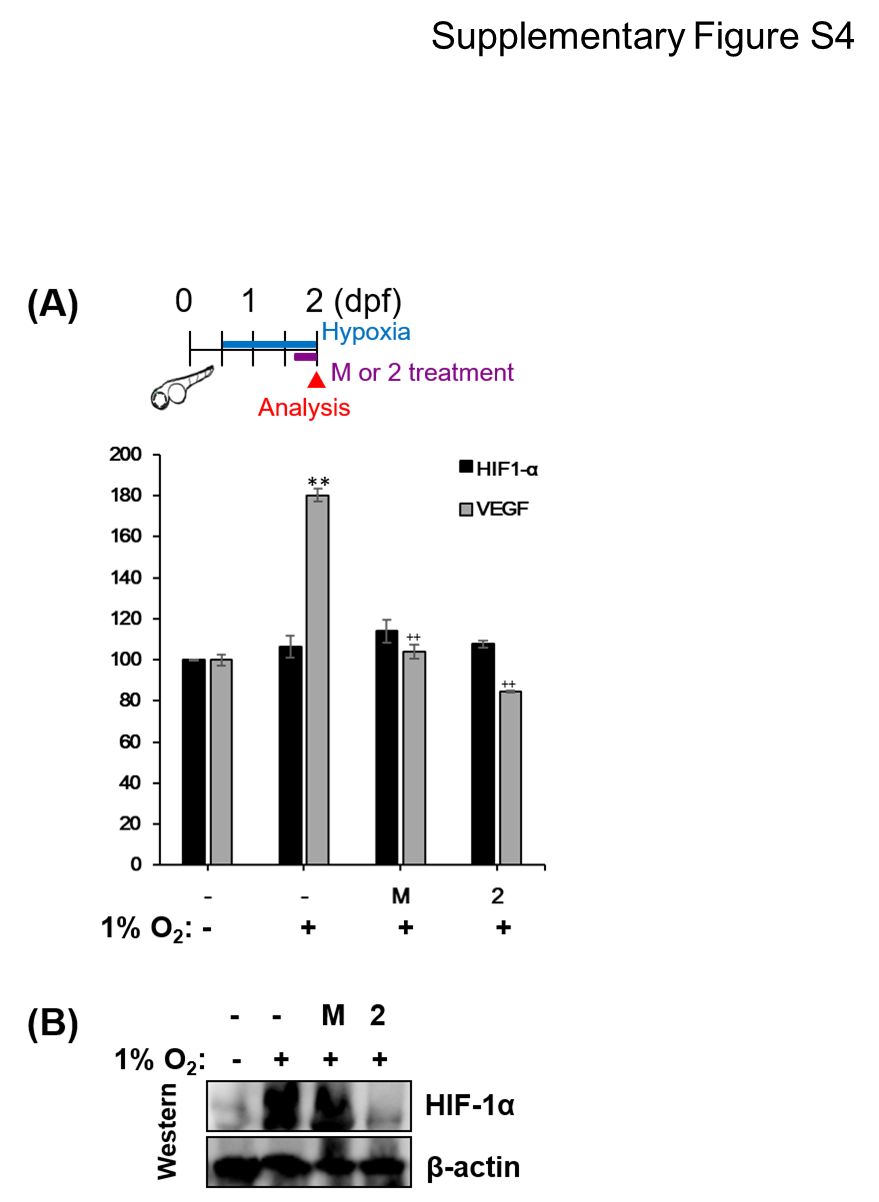
***

***
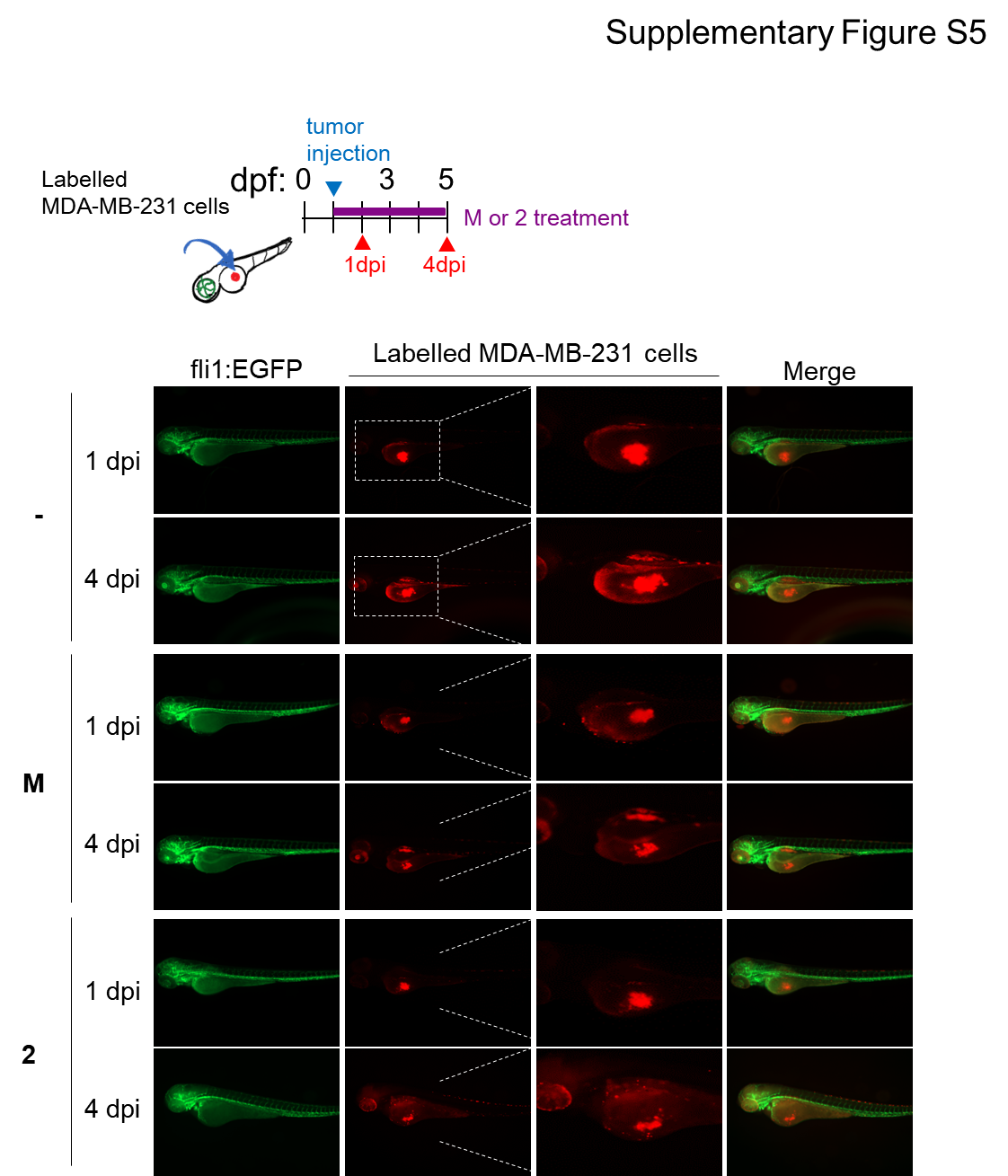
***

***
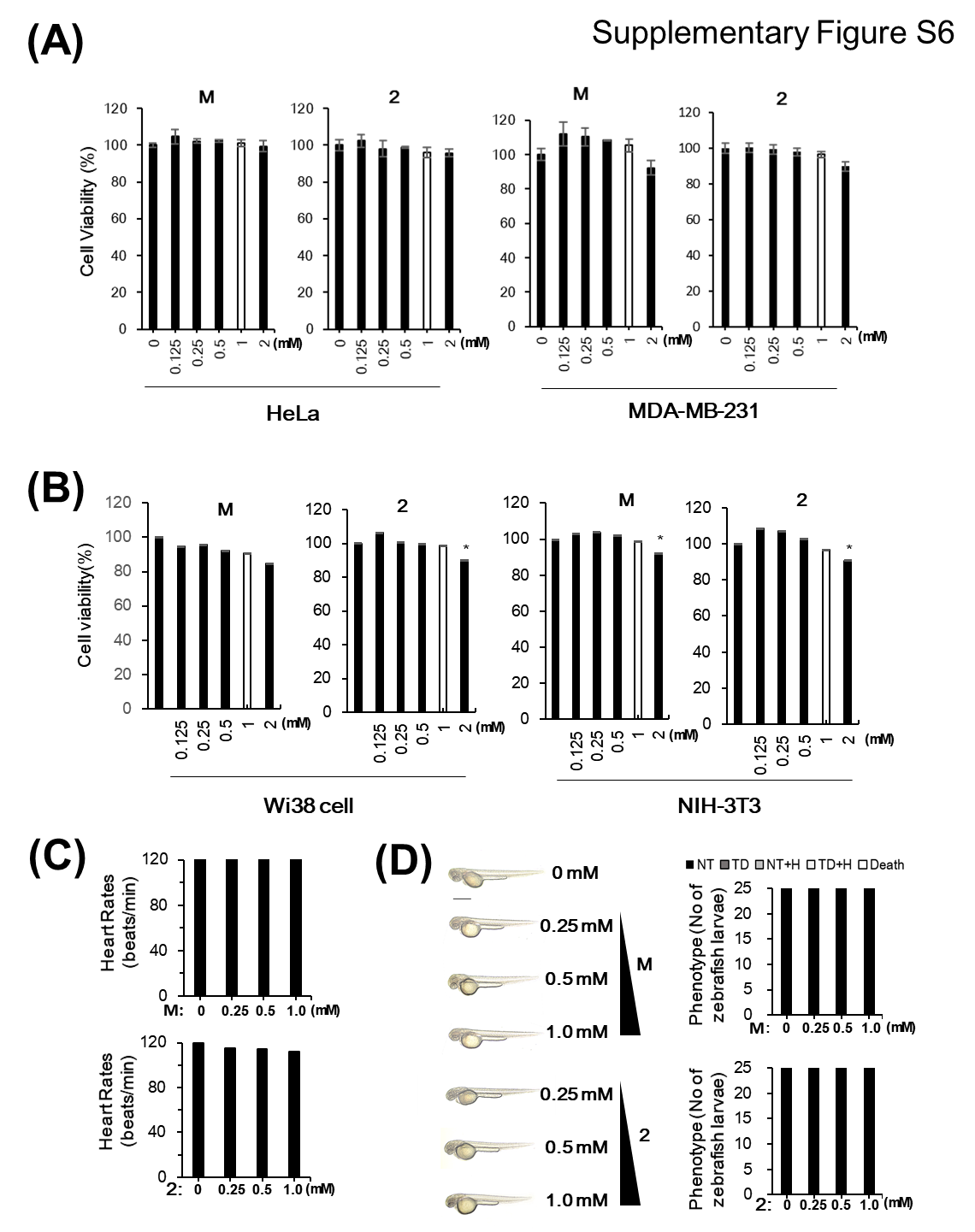
***

***
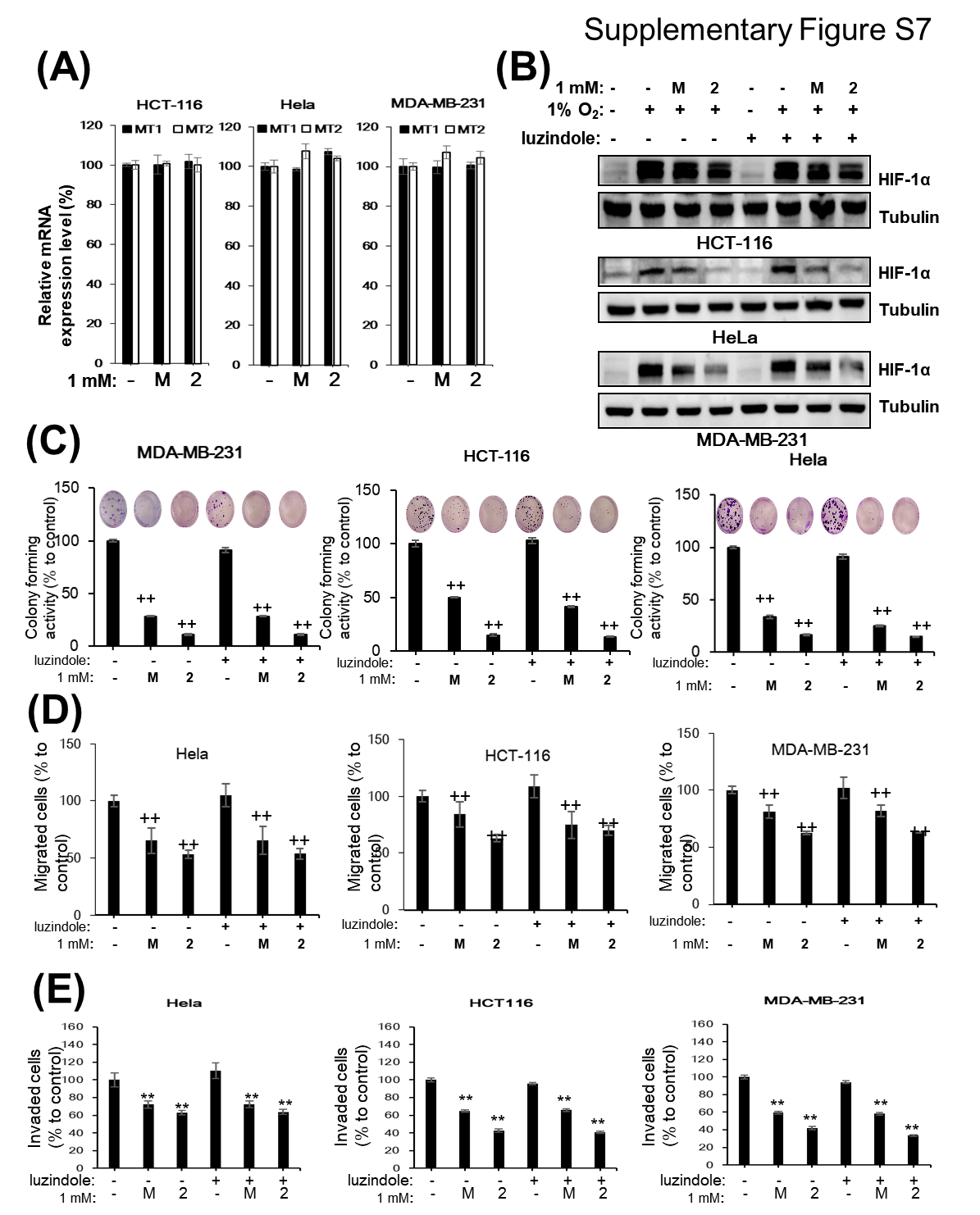
***

**1. Synthetic procedures and characterizations of melatonin and its derivatives**

**Melatonin (M)**

Triethylamine (TEA, 0.33 mL, 2.35 mmol) was added to 5-methoxytryptamine (**1**, 300 mg, 1.57 mmol) in dichloromethane (10 mL). Acetyl chloride (0.017 mL, 2.35 mmol) was added to the reaction mixture and stirred at room temperature for 1 hr. The mixture was diluted with ethyl acetate (40 mL), washed with brine (3×20 mL), dried (MgSO_4_), and concentrated *in vacuo*. The residue was puriﬁed by ﬂash-chromatography (silica gel, hexane:EtOAc = 1:1) and melatonin (**M**) was obtained as white solid (222 mg, 60% yield). mp 117^o^C; HRMS (FAB): calcd for [C_13_H_17_N_2_O_2_]^+^ 233.1290, found: 233.1294; ^1^H-NMR (500MHz, CDCl_3_) δ 6.85‒6.84 (m, 1H), 6.46‒6.44(m, 1H), 3.44 (s, 3H), 3.17 (q, *J* = 6.5, 2H), 2.52 (t, *J* = 6.5, 2H), 1.51 (s, 3H) ppm; ^13^C-NMR (125MHz, CDCl_3_) δ 170.2, 154.0, 131.6, 127.7, 127.73, 122.9, 112.1, 112.4, 100.4, 56.0, 39.6, 39.6, 25.1, 23.4 ppm; IR (ATR) 3301, 3080, 2990, 2927, 2872, 2827, 2359, 1618, 1586, 1553, 1488, 1370, 1211, 1176, 1040, 925, 824, 796, 712 cm^-1^.

***N*-(2-(5-Methoxy-1H-indol-3-yl)ethyl)butyramide (2)**

TEA (0.11 mL, 0.8 mmol) was added to 5-methoxytryptamine (**1**, 100 mg, 0.53 mmol) in dichloromethane (3.0 mL). Butyryl chloride (0.082 mL, 0.8 mmol) was added to the reaction mixture and stirred at room temperature for 1 hr. The mixture was diluted with ethyl acetate (40 mL), washed with brine (3×20 mL), dried (MgSO_4_), and concentrated *in vacuo*. The residue was puriﬁed by ﬂash-chromatography (silica gel, hexane:EtOAc = 1:1) and **2** was obtained as white solid (121 mg, 88% yield). mp 118^o^C; HRMS (CI): calcd for [C_15_H_21_N_2_O_2_]^+^: 261.1603, found: 261.1601; ^1^H-NMR (500 MHz, CDCl_3_) δ 7.26‒7.24 (m, 1H), 7.02‒7.00 (m, 2H), 6.86‒6.85 (m, 1H), 3.84 (s, 3H), 3.60‒3.56 (m, 2H), 2.93‒2.91 (m, 2H), 2.09‒2.06 (m, 2H,), 1.63‒1.58 (m, 2H), 0.91‒0.88 (m, 2H) ppm; ^13^C-NMR (125 MHz, CDCl_3_) δ 173.2, 154.2, 131.7, 127.8, 122.9, 112.9, 112.6, 112.2, 100.6, 56.1, 39.1, 39.7, 25.6, 19.4, 13.9 ppm; IR (ATR) 3262, 2360, 1614, 1560, 1486, 1317, 1217, 1145, 1119, 1073, 974, 922, 825, 794, 667, 640 cm^-1^.

***N*-(2-(5-Methoxy-1H-indol-3-yl)ethyl)benzamide (3)**

TEA (0.11 mL, 0.8 mmol) was added to 5-methoxytryptamine (**1**, 100 mg, 0.53 mmol) in dichloromethane (3.0 mL). Benzoyl chloride (0.092 mL, 0.8 mmol) was added to the reaction mixture and stirred at room temperature for 1 hr. The mixture was diluted with ethyl acetate (40 mL), washed with brine (3×20 mL), dried (MgSO_4_), and concentrated *in vacuo*. The residue was puriﬁed by ﬂash-chromatography (silica gel, hexane:EtOAc = 1:1) and **3** was obtained as white solid (112 mg, 73% yield). mp 103^o^C; HRMS (CI): calcd for [C_18_H_19_N_2_O_2_]^+^: 295.1447, found: 295.1443; ^1^H-NMR (500 MHz, CDCl_3_) δ 7.87‒7.86 (m, 2H), 7.67‒7.64 (m, 1H), 7.59‒7.56 (m, 2H), 7.47‒7.45 (m, 1H), 7.25 (m, 1H), 7.08‒7.05 (m, 1H), 6.49‒6.47 (s, 1H), 4.01‒3.98 (m, 2H), 3.97 (s, 3H), 3.27‒3.25 (m, 2H,) ppm; ^13^C-NMR (125 MHz, CDCl_3_) δ 167.7, 154.2, 134.7, 131.7, 131.6, 128.7, 128.6, 127.0, 123.1, 112.9, 112.8, 112.3, 100.4, 55.9, 40.5, 25.4 ppm; IR (ATR) 3410, 2360, 1870, 1694, 1649, 1533, 1485, 1454, 1323, 1212, 1146, 975, 923, 833, 804, 709, 669, 628 cm^-1^.

***tert*-Butyl (2-(5-methoxy-1H-indol-3-yl)ethyl)carbamate (4)**

TEA (0.11 mL, 0.8 mmol) was added to 5-methoxytryptamine (**1**, 100 mg, 0.53 mmol) in dichloromethane (3.0 mL). Di-*tert*-butyl dicarbonate (183 mg, 0.8 mmol) was added to the reaction mixture and stirred at room temperature for 1 hr. The mixture was diluted with ethyl acetate (40 mL), washed with brine (3×20 mL), dried (MgSO_4_), and concentrated *in vacuo*. The residue was puriﬁed by ﬂash-chromatography (silica gel, hexane:EtOAc = 1:1) and **4** was obtained as white oil (143 mg, 93% yield). HRMS (CI): calcd for [C_16_H_22_N_2_O_3_]^+^: 290.1630, found: 290.1627; ^1^H-NMR (500 MHz, CDCl_3_) δ 7.24‒7.22 (m, 1H), 6.86‒6.85 (m, 2H), 6.84 (m, 1H), 3.85 (s, 3H), 3.45‒3.44 (m, 2H), 2.91‒2.89 (m, 2H), 1.42 (s, 9H) ppm; ^13^C-NMR (125 MHz, CDCl_3_) δ 156.2, 154.1, 131.7, 127.9, 123.0, 113.0, 112.5, 112.1, 100.7, 79.3, 56.1, 40.9, 28.6, 26.0 ppm; IR (ATR) 3333, 2975, 2932, 1845, 1687, 1584, 1510, 1485, 1455, 1392, 1365, 1271, 1249, 1215, 1165, 1072, 956, 922, 855, 795, 637 cm^-1^.

**5-Methoxy-*N*,*N*-dimethyl-1H-indole-2-carboxamide (11)**

Thionyl chloride which was dissolved in dichloromethane (10.46 mL, 10.46 mmol) was added to 5-methoxyindole-2-carboxylic acid (**10**, 1.0 g, 5.23 mmol) in dichloromethane (10 mL). Reaction mixture was under argon for 20 min at room temperature then refluxed for 4 hr. The mixture was evaporated under reduced pressure to remove excess SOCl_2_ and solvents. The residue which was under argon in tetrahydrofuran (THF, 10 mL) was mixed with dimethylamine (9.1 mL, 18.3 mmol) at 0^o^C. Then, the reaction mixture was stirred at room temperature for 16 hr. The mixture was diluted with ethyl acetate (40 mL), washed with brine (3×20 mL), dried (MgSO_4_), and concentrated *in vacuo*. The residue was puriﬁed by ﬂash-chromatography (silica gel, hexane: EtOAc = 1:1) and **11** was obtained as brown solid (923 mg, 81% yield). mp 228^o^C; ^1^H-NMR (500 MHz, CDCl_3_) δ7.35‒7.33 (m, 1H), 7.03 (m, 1H), 6.94‒6.92 (m, 1H), 6.75 (m, 1H), 3.82 (s, 3H), 3.41‒3.20 (m, 6H) ppm.

**1-(5-Methoxy-1H-indol-2-yl)-*N*, *N*, *N* -trimethylmethanaminium (12)**

Lithium aluminium hydride 1.0 M solution in THF (12 mL, 12.0 mmol) was added to compound **11** (1.3 g, 6.0 mmol) in anhydrous THF (15 mL) at 0^o^C under argon. After addition, the reaction mixture was refluxed for 2 hr. To quench excess LiAlH_4_, iced-water was added as same equivalent of LiAlH_4_ until no hydride left. The suspension was filtered on Celite. The filtrated solution was concentrated *in* *vacuo*. The residue was mixed in ethyl acetate (5.0 mL), and methyl iodide (0.4 mL, 6.6 mmol) was added to the mixture. The reaction mixture was refluxed for 1 hr and cooled-down at room temperature for 1 hr. The pale pink solid **12** (1520.4 mg, 73% yield) was collected by glass filtration. mp 173^o^C; ^1^H-NMR (500 MHz, CDCl_3_) δ 7.42‒7.41 (m, 1H), 6.95‒6.91 (m, 2H), 6.56 (s, 1H), 5.25 (s, 2H), 3.80 (s, 3H), 3.27 (s, 9H) ppm.

**2-(5-Methoxy-1H-indol-2-yl)ethan-1-amine (6)**

Compound **12** (900 mg, 2.59 mmol) and potassium cyanide (575 mg, 8.8 mmol) under argon were dissolved in dimethyl sulfoxide (10 mL). The reaction mixture was heated at 80^o^C for 2 hr. The mixture was cooled down with iced-water, extracted with dichloromethane (30×7 mL) and brine (40 mL), dried (MgSO_4_), and concentrated *in vacuo*. The residue was puriﬁed by ﬂash-chromatography (silicagel, hexane:EtOAc = 1:1) and intermediate was obtained as red brown solid (409 mg). To the purified residue (372 mg), ammonia 2.0 M solution in ethanol (6.2 mL, 12.4 mmol) and Raney nickel under hydrogen gas were added in THF (10 mL). The reaction mixture was stirred for 6 hr, filtered and washed with MeOH on Celite. The residue was purified by column chromatography (silica gel, CHCl_3_:MeOH:NH_4_OH = 46:50:4) to afford melatonin-like precursor, **6** as pale yellow solid (327 mg, 72% yield). mp 108‒109^o^C; HRMS (FAB): calcd for [C_11_H_15_N_2_O]^+^: 191.1184, found: 191.1180; ^1^H-NMR (500 MHz, CDCl_3_) δ 7.18‒7.16 (m, 1H), 7.01 (m, 1H), 6.78‒6.76 (m, 1H), 6.17 (s, 1H), 3.83 (s, 3H), 3.04‒3.01 (m, 2H,), 2.83‒2.81 (m, 2H) ppm; ^13^C-NMR (125 MHz, CDCl_3_) δ 154.0, 139.3, 131.0, 129.0, 111.2, 110.9, 101.9, 99.7, 55.9, 41.7, 31.4 ppm; IR (ATR) 3382, 3255, 2520, 2249, 1621, 1556, 1487, 1452, 1379, 1301, 1226, 1197, 1167, 1134, 1026, 975, 941, 834, 794, 756, 724, 660, 622, 587, 517 cm^-1^.

***N*-(2-(5-Methoxy-1H-indol-2-yl)ethyl)acetamide (5)**

TEA (0.11 mL, 0.8 mmol) was added to **6** (100 mg, 0.53 mmol) in dichloromethane (3.0 mL). Acetyl chloride (57 mg, 0.8 mmol) was added to the reaction mixture and stirred at room temperature for 1 hr. The mixture was diluted with ethyl acetate (40 mL), washed with brine (3×20 mL), dried (MgSO_4_), and concentrated *in vacuo*. The residue was puriﬁed by ﬂash-chromatography (silica gel, hexane:EtOAc = 1:1) and **5** was obtained as yellow solid (95 mg, 77% yield). mp 148^o^C; HRMS (FAB): calcd for [C_13_H_16_N_2_O_2_]^+^: 232.1212, found: 232.1205; ^1^H-NMR (500 MHz, CDCl_3_) δ 7.21‒7.19 (m, 1H), 7.00‒6.99 (m, 1H), 6.99‒6.97 (m, 1H), 6.19 (s, 1H), 3.82 (s, 3H), 3.60‒3.56 (m, 2H), 2.96‒2.94 (m, 2H), 1.93 (s, 3H) ppm; ^13^C-NMR (125 MHz, CDCl_3_) δ 170.8, 154.4, 137.2, 131.4, 129.5, 111.6, 111.5, 102.1, 100.5, 56.1, 39.0, 28.9, 23.6 ppm; IR (ATR) 3400, 3149, 2937, 2358, 1652, 1582, 1534, 1456, 1433, 1372, 1318, 1296, 1256, 201, 1164, 1124, 1092, 1049, 1022, 994, 930, 871, 744, 626 cm^-1^.

***N*-(2-(5-Methoxy-1H-indol-2-yl)ethyl)butyramide (7)**

TEA (0.11 mL, 0.8 mmol) was added to **6** (100 mg, 0.53 mmol) in dichloromethane (3.0 mL). Butyryl chloride (0.082 mL, 0.8 mmol) was added to the reaction mixture and stirred at room temperature for 1 hr. The mixture was diluted with ethyl acetate (40 mL), washed with brine (3×20 mL), dried (MgSO_4_), and concentrated *in vacuo*. The residue was puriﬁed by ﬂash-chromatography (silica gel, hexane:EtOAc = 1:1) and **7** was obtained as yellow solid (99 mg, 72% yield). mp 93^o^C; HRMS (FAB): calcd for [C_15_H_20_N_2_O_2_]^+^: 260.1525, found: 260.1530; ^1^H-NMR (500 MHz, CDCl_3_) δ 7.20‒7.18 (m, 1H), 6.99 (m, 1H), 6.78‒6.77 (m, 1H), 6.18 (s, 1H), 3.82 (s, 3H), 3.61‒3.57 (m, 2H), 2.96‒2.93 (m, 2H), 2.11‒2.08 (m, 2H), 1.64‒1.57 (m, 2H), 0.90‒0.87 (m, 3H) ppm; ^13^C-NMR (125 MHz, CDCl_3_) δ 173.5, 154.2, 137.0, 131.2, 129.1, 111.4, 111.3, 101.9, 100.3, 55.9, 38.7, 38.6, 28.9, 19.1, 13.7 ppm; IR (ATR) 3380, 3227, 3059, 2963, 1639, 1588, 1487, 1446, 1351, 1320, 1280, 1204, 1171, 1136, 1107, 1032, 988, 944, 842, 792, 763, 736, 702, 671, 632 cm^-1^.

***N*-(2-(5-Methoxy-1H-indol-2-yl)ethyl)benzamide (8)**

TEA (0.11 mL, 0.8 mmol) was added to **6** (100 mg, 0.53 mmol) in dichloromethane (3.0 mL). Benzoyl chloride (0.092 mL, 0.8 mmol) was added to the reaction mixture and stirred at room temperature for 1 hr. The mixture was diluted with ethyl acetate (40 mL), washed with brine (3×20 mL), dried (MgSO_4_), and concentrated *in vacuo*. The residue was puriﬁed by ﬂash-chromatography (silica gel, hexane:EtOAc = 1:1) and **8** was obtained as yellow solid (139 mg, 87% yield). mp 172^o^C; HRMS (FAB): calcd for [C_18_H_18_N_2_O_2_]^+^: 294.1368, found: 294.1367; ^1^H-NMR (500 MHz, CDCl_3_) δ 7.69‒7.67 (m, 2H), 7.48‒7.46 (m, 1H), 7.40‒7.37 (m, 2H), 7.20‒7.19 (m, 1H), 7.00 (m, 1H), 6.79‒6.77 (m, 1H), 6.24 (s, 1H), 3.82 (s, 3H), 3.80‒3.77 (m, 2H), 3.08‒3.05 (m, 2H) ppm; ^13^C-NMR (125 MHz, CDCl_3_) δ 168.1, 154.4, 137.1, 134.4, 131.9, 131.5, 130.4, 129.3, 128.9, 128.7, 127.1, 111.6, 111.6, 102.1, 100.6, 56.1, 39.6, 29.1 ppm; IR (ATR) 3417, 3240, 1698, 1640, 1577, 1522, 1484, 1442, 1407, 1284, 1222, 1193, 1163, 1139, 1028, 940, 838, 808, 774, 706 cm^-1^.

***tert*-Butyl (2-(5-methoxy-1H-indol-2-yl)ethyl)carbamate (9)**

TEA (0.11 mL, 0.8 mmol) was added to **6** (100 mg, 0.53 mmol) in dichloromethane (3.0 mL). Di-*tert*-butyl dicarbonate (183 mg, 0.8 mmol) was added to the reaction mixture and stirred at room temperature for 1 hr. The mixture was diluted with ethyl acetate (40 mL), washed with brine (3×20 mL), dried (MgSO_4_), and concentrated *in vacuo*. The residue was puriﬁed by ﬂash-chromatography (silica gel, hexane:EtOAc = 1:1) and **9** was obtained as yellow solid (131 mg, 85% yield). mp 74^o^C; HRMS (FAB): calcd for [C_16_H_22_N_2_O_3_]^+^: 290.1630, found: 290.1625; ^1^H-NMR (500 MHz, CDCl_3_) δ 7.19‒7.14 (m, 1H), 7.00 (m, 1H), 6.78‒6.76 (m, 1H), 6.19 (s, 1H), 3.82 (s, 3H), 3.47‒3.45 (m, 2H), 2.94‒2.92 (m, 2H), 1.41 (s, 9H) ppm; ^13^C-NMR (125 MHz, CDCl_3_) δ 156.3, 154.2, 137.4, 131.4, 129.2, 111.5, 111.3, 102.0, 100.3, 79.8, 56.0, 40.0, 29.2, 28.5 ppm; IR (ATR) 3679, 3375. 2979, 2865, 1680, 1662, 1589, 1521, 1484, 1452, 1366, 1287, 1251, 1226, 1199, 1165, 1135, 1113, 1055, 1032, 942, 863, 835, 806, 775, 653, 616, 591, 552, 517 cm^-1^.

**2. Materials and method for additional biological assay**

**Co-immunoprecipitation (Co-IP)**

Hypoxia-inducible factor-1α and pVHL were immunoprecipitated from 1.0 mg of cell lysates from HCT116 cells. Cell lysates were incubated with 1.0 μg of anti-rabbit HIF-1α antibody (Santa Cruz Biotechnology) in a total volume of 500 μL of ice-cold lysis buﬀer [50 mM Tris-Cl (pH 7.5), 150 mM NaCl, 1% Nonidet P-40, 10% glycerol]. After rocking for 24 hr at 4^o^C, 20 μL of protein A-Sepharose beads (1:1 slurry; Gibco BRL, USA) was added. The mixtures were incubated overnight at 4^o^C. The precipitates were boiled with an equal volume of 5X LSB at 95^o^C for 3 min. The eluted proteins were separated on 8% SDS-polyacrylamide gel and detected by Western blot analysis with anti-human HIF-1α (BD Biosciences) and anti-human pVHL (BD Biosciences).

**Reverse transcription polymerase chain reaction (RT-PCR) and real-time PCR**

RT-PCR and real-time PCR were done as described previously [S1]. Briefly, total RNA from HCT116 cells or zebrafish larvae was purified by Trizol Reagent (Invitrogen Corporation, USA) and cDNA was synthesized by M-MLV reverse transcriptase (Promega, USA) according to the manufacturer’s instructions. For real-time PCR, VEGF165 and HIF-1α primers were used as followed. Forward strand VEGF165 5’-GAAAGAAAACCACTGTGAGCCT-3’, reverse strand VEGF165 5’-TCGCTCGATCATCATCTTGGC-3’, forward strand HIF-1α 5’-CTACAATGAT-GTCATGCTGCC-3’, reverse strand HIF-1α 5’-ACACAGAGTGAGTGGCAGAA-3’. Quantitative real-time PCR was performed in triplicate on Rotor-Gene® Q (Roche Diagnostics, USA) using LightCycler® SYBR-Green I Master (Roche Diagnostics), and data were analyzed on the basis of threshold cycle values of each sample and normalized with β-actin

**Transient transfection and luciferase assays**

Dual luciferase reporter assays were performed using the dual luciferase kit (DLR) from Promega and pSV40promoter-EpoHRE-Luc, pBOS-HIF-1α and pBOS-ARNT plasmid constructs, respectively. The reporter plasmid and a pTK-Renilla (transfection normalization vector, Promega) were mixed (10:1) before dilution (1.6%) in OptiMEM (Invitrogen). Cells were transfected using a Lipofectamine plus transfection reagent (Invitrogen) for assessing HIF-1 activity.

**Mouse xenograft model of melanoma B16F10 cells**

B16F10 cells (3 × 10^6^ cells/mL, 100 μL) were injected subcutaneously into the left flank of C57BL/6 mice and injected mice were randomly subdivided into three groups (7 of each group). After 7 days, 10 mg/kg of melatonin (**M**) or NB-5-MT (**2**) was injected intraperitoneally every day for a week. Normal saline was treated as a negative control. Then, mice were sacrificed and the implanted melanomas were separated and used for immunohistochemistry.
