# Supplementary material for: Enhanced anti‐angiogenic activity of novel melatonin‐like agents
Source: J Pineal Res. 2021 May 13;71(1):e12739. doi: 10.1111/jpi.12739 (PMC8365647; doi:10.1111/jpi.12739)
Supplement: Supplementary file 2 — Supplementary Material [file JPI-71-e12739-s002.docx]

**Supplementary Figure legends**

**Supplementary Fig. S1** Synthesis of (A) melatonin (**M**) and its derivatives **2**‒**4** from **1** [Reaction conditions: a) RCOX, Et_3_N, CH_2_Cl_2_, room temperature (rt), 1 hr]; (B) derivatives **5**‒**9** from **10** [Reaction conditions: a) SOCl_2_, CH_2_Cl_2_, reflux, 4 hr, b) NH(CH_3_) _2_, tetrahydrofuran (THF), rt, 16h, 81%, c) LiAlH_4_, THF, reflux, 2 hr, d) CH_3_I, EtOAc, reflux, 2 hr, 73%, e) KCN, dimethyl sulfoxide (DMSO), 80^o^C, 2 hr, f) NH_3_/EtOH, Raney Ni/H_2_, THF, 60 ^o^C, 6 hr, 72%, g) RCOX, Et_3_N, CH_2_Cl_2_, rt, 1 hr].

**Supplementary Fig. S2 Inhibitory effect of NB-5-MT(2) on the mRNA expression levels of HIF-1-α and its target genes in HeLa and MDA-MB-231 cells.** (A) The effect **2** on HIF1α, VEGF, Glut1, and EPO mRNA expression under hypoxic conditions in HeLa cells. (B) The effect **2** on HIF1α, VEGF, Glut1, and EPO mRNA expression under hypoxic conditions in MDA-MB-231 cells.

**Supplementary Fig. S3 Log *P*-values of melatonin-like molecules.** Most melatonin derivatives (except **5** and **6**) showed higher log *P*-values than melatonin (log *P*_melatonin_ = 0.71), suggesting that these derivatives had better permeability.

**Supplementary Fig. S4 NB-5-MT (2) suppresses HIF-1α expression in zebrafish larvae under hypoxia.** Under hypoxia, zebrafish larvae were treated with either melatonin (**M**) or **2** for 4 hr. (A) Inhibitory effect of **2** on VEGF and HIF-1α mRNA expression in zebrafish larvae under hypoxia. (B) HIF-1α expression was examined by western blot analysis.

**Supplementary Fig. S5 NB-5-MT (2) inhibits tumor growth in a zebrafish xenograft model *in vivo*.** Human MDA-MB-231 cancer cells were injected into the abdominal perivitelline space of Tg(fli1:EGFP) zebrafish larvae at 2 days postfertilization (dpf) and treated with vehicle, melatonin (**M**), or NB-5-MT (**2**). Photographs were taken at 1 and 4 dpi, showing the changes in xenograft size and the number of cancer cells that invaded into nearby tissues. EGFP expression indicates the entire vasculature under the control of the fli1 promoter.

**Supplementary Fig. S6 Toxicity analysis of NB-5-MT (2) *in vitro* and *in vivo*.**

(A) The effect of NB-5-MT (**2**) on cell viability in HeLa cervical cancer cells and MDA-MB-231 breast cancer cells. (B) The effect of **2** on cell viability in normal cells, Wi38 lung fibroblast cells and NIH-3T3 embryonic fibroblasts. White bars indicate the cell viability when treated with compound at 1.0 mM. Values are expressed relative to vehicle-treated cells, normalized to 100% ± the standard deviation (S.D.), and were obtained from 3 independent experiments. **P* < 0.05 in vehicle vs. compound treatment. (C) Heart toxicity of **2**. Zebrafish larvae were exposed to different concentrations (0.125–2 mM) of **M** and **2** between 24 hpf and 48 hpf. The heart rate was measured by observing heartbeat under a microscope. (D) Phenotype distribution after **2** treatment. Zebrafish larvae were exposed to different concentrations (0.25–2 mM) of **M** and **2** between 24 hpf and 48 hpf. The developmental defects were assessed by phenotype distribution such as survival and morphological changes using a stereomicroscope coupled with a digital camera (Nikon). The representative images (right) of the zebrafish larvae.TD; trunk deformity, H; hemorrhage, NT; normal trunk.

**Supplementary Fig. S7 Involvement of melatonin receptors in the inhibitory effect of NB-5-MT(2).** (A) The effect of **M** and **2** on melatonin receptor 1 (MT1) and MT2 mRNA expression levels in HCT116, HeLa, and MDA-MB-231 cells. (B) The effect of 20 μM luzindole (a well-known MT1/MT2 antagonist) on **2**-mediated HIF-1 downregulation in HCT116, HeLa, and MDA-MB-231 cells. Under hypoxia, cells were incubated with or without 1.0 mM of **2** in the presence of the luzindole for 24 hr. (C) The effect of 20 μM luzindole on **2**-mediated downregulation of colony formation in HCT116, HeLa, and MDA-MB-231 cells. The representative images (right) of the colony. (D) The effect of 20 μM luzindole on **2**-mediated downregulation of migration in HCT116, HeLa, and MDA-MB-231 cells. (E) The effect of 20 μM luzindole on **2**-mediated downregulation of invasion in HCT116, HeLa, and MDA-MB-231 cells.
